# *Zanthoxylum* Species: A Comprehensive Review of Traditional Uses, Phytochemistry, Pharmacological and Nutraceutical Applications

**DOI:** 10.3390/molecules26134023

**Published:** 2021-06-30

**Authors:** Innocent Uzochukwu Okagu, Joseph Chinedu Ndefo, Emmanuel Chigozie Aham, Chibuike C. Udenigwe

**Affiliations:** 1 Department of Biochemistry, University of Nigeria, Nsukka 410001, Enugu State, Nigeria; innocent.okagu@unn.edu.ng (I.U.O.); emmanuel.aham@unn.edu.ng (E.C.A.); 2 Department of Science Laboratory Technology, University of Nigeria, Nsukka 410001, Enugu State, Nigeria; 3 Natural Science Unit, School of General Studies, University of Nigeria, Nsukka 410001, Enugu State, Nigeria; 4 School of Nutrition Sciences, University of Ottawa, Ottawa, ON K1H 8M5, Canada

**Keywords:** *Zanthoxylum* species, nutraceuticals, ethnobotanical use, phytochemicals, biological activities

## Abstract

*Zanthoxylum* species (Syn. *Fagara* species) of the *Rutaceae* family are widely used in many countries as food and in trado-medicinal practice due to their wide geographical distribution and medicinal properties. Peer reviewed journal articles and ethnobotanical records that reported the traditional knowledge, phytoconstituents, biological activities and toxicological profiles of *Z.* species with a focus on metabolic and neuronal health were reviewed. It was observed that many of the plant species are used as food ingredients and in treating inflammation, pain, hypertension and brain diseases. Over 500 compounds have been isolated from *Z.* species, and the biological activities of both the plant extracts and their phytoconstituents, including their mechanisms of action, are discussed. The phytochemicals responsible for the biological activities of some of the species are yet to be identified. Similarly, biological activities of some isolated compounds remain unknown. Taken together, the *Z.* species extracts and compounds possess promising biological activities and should be further explored as potential sources of new nutraceuticals and drugs.

## 1. Introduction

Plants have provided useful sources of nutrients and therapeutic preparations for managing several health conditions in many parts of the world. The vast majority of people who use plant extracts as medicine consider them safer compared to synthetic drugs [1]. In addition, many types of infections, parasitic diseases, and tumors that are resistant to some synthetic drugs have been shown to be treatable using herbal medicines [1,2,3,4,5]. Furthermore, phyto-bioactive compounds have been shown to provide templates that direct the development of some drugs in current use, such as quinine and artemisinins for malaria, and alkaloids and camptothecins for cancer. *Zanthoxylum* Linn. is a plant genus belonging to Rutaceae, a family that includes over 160 genera and 2000 species with more species still being identified [6]. Plant species in this genus are distributed all over the world with the majority found in Asia, America and Africa [7,8,9,10,11]. Many *Z.* species are traditionally used as medicinal plants in managing various health conditions. Secondary metabolites isolated from parts of plant species in this genus have demonstrated several pharmacological activities, such as antioxidant, analgesic, anti-inflammatory activities, and modulatory effects against obesity, dementia, and diabetes [12,13,14,15]. Traditionally, decoctions of *Z.* species*,* either as a single plant-based preparation or in combination with other plants, are commonly used in treating infection of various kinds, especially those caused by parasites (malaria, leishmaniasis, trypanosomiasis, and other parasitic diseases), sickle cell, tumor, bacterial, fungal, and viral infections [16,17,18,19,20,21]. They are also used to repel insects especially those carrying vector-borne diseases, like mosquitoes, and hence are beneficial in preventing vector-borne diseases [22].

Different aspects of *Z.* species have been previously reviewed by Negi et al. [23], Singh and Singh [24], Brijwal et al. [25], Mukhtar and Kalsi [26], and Paul et al. [27] for *Z. armatum,* Supabphol and Tangjitjareonkun [22] for *Z. limonella,* Zhang et al. [28] for *Z. bungeanum,* Sinan et al. [29] for *Z. gilletii*, Guendéhou et al. [30] for *Z. zanthoxyloides*, and Lu et al. [31] for *Z. nitidum*. The present review provides comprehensive analysis and discussion covering mostly studies that were not captured in the previous reviews. Specifically, this review summarizes the ethnobotanical reports and biological activities of over 39 *Z.* species. Specifically, pharmacological potentials of *Z.* species in relation to metabolic health, such as antioxidant, analgesics, anti-inflammatory, and modulatory effects against markers of organ damages, ulcer, obesity, dementia, and diabetes, were discussed. In addition, isolated secondary metabolites from these species are presented. It is hoped that the outcome of this review will position plant species of this genus as sources of nutraceutical and medicinal compounds while spurring more research on other plants in this genus used in traditional medicine.

## 2. Traditional Uses of *Zanthoxylum* Species as Food and Medicine

The traditional uses of *Zanthoxylum* species as food and in medicinal practices are highlighted in this section. In South Africa and Kenya, pastes made from *Z.* species are used to suppress pain associated with wounds and to aid wound healing [32,33] while in Nigeria, *Z.* species like *Z. zanthoxyloides* are used for treating rheumatism, sickle cell anemia [34,35], toothache, urinary tract infection, and venereal diseases [36]. Similarly, *Z. zanthoxyloides* root-bark is used in Uganda for healing elephantiasis, toothache, erectile dysfunction, gonorrhea, malaria, dysmenorrhea, and abdominal pain [21,22]. Stem decoction of *Z. zanthoxyloides* is used in Cote D’Ivoire to relieve tooth pain and to treat infections caused by oral pathogens [37]. In addition, *Z. zanthoxyloides* leaves are used in Togo to treat wounds, root-bark for toothache, swellings, and worms and to induce lactation post-partum, while the bark is used for relieving pain [38]. In addition, the stem bark decoction is used in treating malaria in Ghana [8]. In Central African Republic, different parts of *Z. zanthoxyloides* and *Z. clava-herculis* L. (Syn. *Z. macrophyllum* Nutt.) are traditionally used for healing diseases of the circulatory and respiratory systems, malaria, diabetes, and hypertension [39]. 

In Côte D’Ivoire, *Z. gilletii* is widely used to treat malaria [40], skin infections, and hypertension [41]. In other parts of Africa, decoctions from different parts of *Z. gilletii* are also used either alone or in combination to treat oral diseases and erectile dysfunction [42], female reproductive issues [43], rheumatism and many kinds of pains [44,45]. According to Kipkore et al. [45], *Z. chalybeum* bark and seeds are boiled and consumed to relieve pain associated with surgeries and other forms of pain, and to treat malaria and amoebiosis, while burnt ashes from the bark and seeds are used to treat rheumatism. Additionally, stem bark of *Z. chalybeum* Engl., bark of *Z. gilletii* and root, fruits, bark, leaves, and stem of *Z. usambarense* are used alone or in combination in Kenya to treat malaria and related symptoms like fever [46]. Aerial parts and barks of *Z. fagara* (L.) Sarg. (Syn. Z. *affine* Kunth and *Z. hyemale* A. St.-Hil.), *Z. elephantiasis* Macfad. and *Z. martinicense* (Lam.) DC.) are traditionally used in Cuba in treating diarrhea, heart diseases, fever, and many types of pain such as ear and muscle, and toothaches [47]. Moreso, aerial parts of *Z. acanthopodium* DC. are applied traditionally in China as contraceptives, in suppressing pain and in parasite control [48]. In South Africa, roots of *Z. capense* (Thunb.) Harv. in combination with *Callilepis laureola* root are taken as oral preparation for respiratory tract and oral infections [49]. Similarly, the Zulu people of South Africa traditionally use bark and root bark decoction of *Z. capense* for treating tuberculosis [50]. In Thailand, *Z. rhetsa* (Syn. *Z. budrunga*) is used as spices and condiments for cooking, as well as for treating infections [1]. In Cameroon and Madagascar, stem bark of *Z. gilletii* is traditionally applied in treating hypertension and related disorders [51], microbial infection, cancer, and inflammation [3], while the stem bark of *Z. tsihanimposa* H. Perrier is traditionally used in Madagascar for treating inflammation, skin diseases, microbial infection, and malaria [52]. 

In general, findings from the articles reviewed show that many *Z.* species are widely used as food and medicine, in Africa, and different parts of the world for treatment of several diseases. In some cases, different populations use the same plant for different conditions, although the traditional uses of most species in traditional medicine are similar in different parts of the world. The ethnobotanical uses of some *Z.* species are summarized in Table 1.

## 3. Phytochemical Constituents of *Zanthoxylum* Species

**Figure 1:**
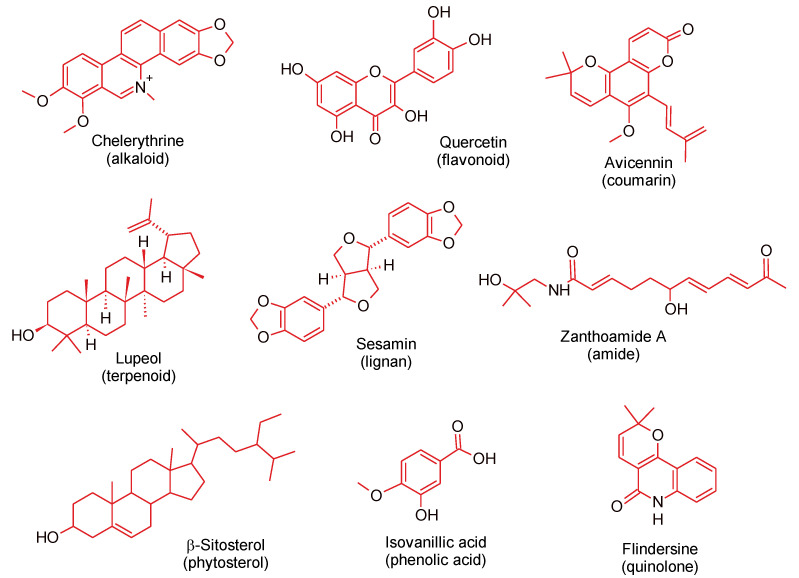
Representative compounds from the different classes of phytochemicals isolated from *Zanthoxylum* species.

The biological activities and toxicity of plants are largely dependent on the composition of their secondary metabolites, also known as phytochemicals. Generally, phytochemicals are non-nutritive compounds synthesized by plants in response to external biotic and abiotic factors. Several classes of phytochemicals have been detected in *Zanthoxylum* species, such as terpenes, flavonoids, coumarins, phenolic acids, and alkaloids, the most reported among all the classes [99,100,101,102]. In this section, we present only the compounds isolated from different parts of *Z.* species whose biological activities are related to metabolic and neuronal health. Representative compounds from each of the classes are shown in Figure 1.

In *Z*. *zanthoxyloides,* neohesperidin, hesperidin, and quercetin were found to be higher in the root and trunk extracts than in the stem and leaf extracts; hyperoside, quercetin-3-*O*-glucopyranoside, datiscin, and quercitrin were found to be higher in the leaf extract, while eriocitrin was found in the fruit extract only, and this may explain why the roots have stronger antioxidant properties [103]. Moreover, Tine et al. [104] analyzed volatile compounds in oils from *Z. zanthoxyloides* and found that the leaf oil is rich in hexadecanoic acid, germacrene D and decanal while pellitorine is the major content of the root and stem bark. Thus, the leaf essential oil is a rich source of antioxidant volatile compounds. Other antioxidant compounds isolated from *Z. zanthoxyloides* include atanine, hesperetin, isoplatydesmine, *N*-methylplatydesminium cation, myrtopsine, ribalinine, *N*-methylatanine, *trans*-fagaramide, zanthoamides G-I, (+)-sesamin, skimmianine and hesperidin from the fruits [58]; 4′-(4′′-hydroxy-3′′-methylbutyloxy)-2-phenylethanol, hydrocuspidiol, cuspidiol, 4′-(3′′-methylbut-2′′-enyloxy)-3-phenylpropanol, dihydrocusidiol, lupeol, 8-acetonyldihydrochelerythrine, N-isobutyl-(2*E*,4*Z*)-deca-2,4-dienamide, (+)-sesamin [62], burkinabins A, B and C, N,N-dimethyllindicarpin, 1,8-di-O-(3-methoxy-4-hydrobenzoyl)-3,6-dihydroxycyclooctane-2,7-endoperoxide, hesperidin, fagaronine, and norchelerythrine [105] from the root bark; and flavonoids (rutin and quercetin) and phenolic acids (caffeic and chlorogenic acids) as the major constituents of the stem bark [106].

The similarity in phytochemical constituents of *Z.* species is demonstrated by the existence of alkaloids such as 8-acetonyldihydrochelerythrine in *Z. paracanthum* Kokwaro stem bark [73], *Z. zanthoxyloides* root bark [105] and *Z. gilletii* stem bark [107]. Futhermore, terpenoides such as lupeol was also reported in *Z. sprucei* Engl. stem bark [90] and *Z. gilletii* stem bark [107,108]. In addition, monoterpenes (myrcene, limonene, and camphene) were reported in essential oils of *Z. gilletti* leaves, alkaloids (peroxysimulenoline, sanguinarine, xanthoplanine, fagarine I and norchelerythrine) in the bark, root and leaf extracts of *Z. zanthoxyloides,* *Z. bungeanum* and *Z. gilletii* multiple biological activities [44,109]. Also, flavonoides such as quercetin and hesperidin were detected in leaves, fruits and root bark of *Z. zanthoxyloides* [58,103,105] and in *Z. sprucei* stem bark (Table 2) [90]. Going further, lignans such as sesamin were reported in *Z. nitidum* root [56], *Z. zanthoxyloides* fruits [58] and *Z. nitidum* stem bark [110]. Based on the above reports, it can be inferred that the existence of similar chemical compounds with multiple biological activities in different parts of *Z.* species may explain the relatedness in biological activities and the multifunctionality of extracts of these plants in relation to metabolic health [63,66,110].

Based on class of compound and biological activity, phytochemicals present in different parts of *Z.* species with metabolic health-promoting properties are summarized in Table 2 (alkaloids), Table 3 (flavonoids), Table 4 (coumarins and derivatives), Table 5 (terpenes and derivatives), Table 6 (lignans and derivatives), Table 7 (amides), Table 8 (phytosterols and derivatives, phenol and phenolic acids, tannins, fatty acids, phenylpropanoids and steroids), and Table 9 (other classes of compounds).

Some of the biological properties of chemicals isolated from these plants were discussed in the following sections. Other phytochemical constituents of plants in this genus are discussed in previous reviews [10,95,136,137] and were hence excluded.

## 4. Health-Related Bioactive Properties of *Zanthoxylum* Species

Several biological activities have been reported for extracts and isolated compounds from genus *Zanthoxylum* species mostly in relation to their traditional uses in different parts of the world. The biological activities in relation to neuronal and metabolic health, such as antioxidant, analgesic, chemoprotective, antidiabetic, antiulcer, anti-Alzheimer’s disease, anti-inflammatory, and anti-hypertensive activities are discussed in this section.

### 4.1. Antioxidant Activities of Zanthoxylum Species 

The involvement of oxidative stress in the development and progression of many diseases has been well-characterized. The pathogenesis of neurodegenerative and cardiovascular diseases, many cancers, and malaria involve oxidative stress [138]. Nature has equipped living cells with enzymatic and non-enzymatic antioxidant defense mechanisms to prevent oxidative damage to cellular components [139,140]. However, humans are exposed to large amount of oxidants, warranting additional support with exogenous antioxidants. The role of dietary antioxidants in the prevention and management of many chronic diseases, such as stroke, atherosclerosis, and cancer is increasingly being recognized [141,142,143,144]. Plant-originated compounds may serve as antioxidants to modulate symptoms associated with disease conditions and to improve the quality of life [145]. Many plant-derived compounds have undergone clinical trials for managing chronic diseases and some of their effects are based on antioxidant mechanisms.

Two compounds isolated from *Z. bungeanum*—magnoflorine from the pericarp and arbutin from the seeds—were shown to have poor antioxidant activities [146]. Considering the traditional use of the fruits as food condiment, Yamazaki et al. [33] isolated hyperoside and quercitrin from *Z. bungeanum* fruits, which strongly inhibited lipid peroxidation and scavenged free radicals in vitro.

In addition, crude methanol extract of *Z. armatum* leaves, its solvent fractions and essential oils were reported to demonstrate good radical scavenging, ferric reducing, and divalent metal chelating potentials, which were directly proportional to the total phenolic content of the samples. The authors further showed that ethyl acetate fraction of the extract had higher metal chelating properties while the essential oil has the highest reducing power [85]. Similarly, Negi et al. [123] reported that *Z. armatum* leave essential oil (containing bornyl acetate, cymene, α-copaene, γ-terpinene, camphene, limonene, linalool, β-ocimene, *trans*caryophyllene, α-terpinolene and germacrene as the most abundant constituents) exhibited strong 2,2′-diphenyl-1-picrylhydrazyl (DPPH) radical scavenging activity (IC_50_ = 27 μg/mL) relative to activity of ascorbic acid (IC_50_ = 15.0 μg/mL).

Moreover, Imaga et al. [147] showed that *Z. zanthoxyloides* root extracts had potent antioxidant and anti-sickling effects. This is further supported by Tine et al. [103] who reported that methanol extracts of *Z*. *zanthoxyloides* fruits, leaves, stems, trunk barks, and root barks possessed antioxidant properties with leaf and trunk bark extracts showing better activities.

In addition, polyphenol-rich crude ethanol extract of *Z. budrunga* seeds showed DPPH radical scavenging activity (IC_50_ = 82.60 μg/mL) compared to ascorbic acid (IC_50_ = 12.58 μg/mL) [131]. Furthermore, crude ethanol extract of *Z. syncarpum* Tull. branches and its alkaloidal fraction inhibited DPPH radical scavenging activities with IC_50_ values of 140 μg/mL and 56 μg/mL, respectively, relative to that by quercetin (IC_50_ = 4.77 μg/mL); the weak antioxidant activity of the extract is attributed to its low total phenolic content (12.43 mg GAE/g). The crude extract and alkaloidal fraction also potently inhibited hydrochloric acid-induced corrosion, with the alkaloidal fraction being more active than the crude extract. An amino compound, 4-(methylamino)-benzoic acid (Table 9) isolated from the alkaloidal fraction showed the best corrosion inhibitory activity, suggesting that it might be responsible for the corrosion prevention activity of the plant extracts [148].

Similarly, Tatiana et al. [15] compared the antioxidant activities of methanol and aqueous extracts of *Z. leprieurii* stem bark and found that the methanol extract had higher antioxidant activities, showing that organic solvent was a better solvent for extracting the antioxidant phytochemicals in the plant stem bark. Taken together, these studies demonstrate that *Z.* species have good antioxidant effects that can be harnessed for prevention and treatment of oxidative stress-related conditions. As shown in Table 2, Table 3, Table 4, Table 5, Table 6, Table 7, Table 8 and Table 9, many antioxidant compounds have been isolated from several *Z.* species, thus justifying the versatile traditional use of the species in disease management and treatment. Furthermore, future research should explore the antioxidant properties of these compounds in preventing rancidity of oil-based food, and their molecular cellular mechanisms for use as plant-derived nutraceuticals.

### 4.2. Neuroprotective and Alzheimer’s Disease Modulatory Effects of Zanthoxylum Species 

Alzheimer’s disease (AD) and its major symptom, dementia, is prevalent in older people, usually above 70 years old, but also in some younger individuals. This trend is related to the growing number of people with AD risk factors, such as obesity, sustained hyperglycemia, high blood pressure, physical inactivity, and metabolic syndrome [148]. An estimate of more than 120,000 deaths associated with AD was recorded in the US in 2018; this places AD as the fifth leading cause of death in those 65 and above [149]. According to GBD 2016 Alzheimer’s Disease and Other Dementia Collaborators [150], the number of people living with AD increased by 53.9% between 1990 and 2016; it is estimated that this number will double by 2040 [130]. There is a significant socio-economic burden associated with AD, thus the need for a concerted effort to explore new ways of reducing the disease incidence. Considering the involvement of oxidative stress in the pathogenesis of AD, research is needed to fully understand the role and molecular basis antioxidants in preventing and managing the disease and its symptoms. Cholinesterase inhibitors are currently used for the management of AD due to its positive effect in suppressing dementia and other symptoms of the condition. Plant-derived bioactive compounds with cholinesterase inhibitory activities are gaining relevance as potential source of lead compounds for AD.

Wang et al. [126] isolated nine alkylamides, including tetrahydrobungeanool, zanthoamides E and F, ZP-amides A-E and (2*E*,7*E*,9*E*)-*N*-(2-hydroxy-2-methylpropyl)-6,11-dioxo-2,7,9-dodecatrienamide, from *Z. bungeanum* pericarps. In vitro neuritogenic test using rat pheochromocytoma PC-12 showed that the nine compounds enhanced neurite outgrowth, indicating that they have neuritogenic activity, without apparent cytotoxicity. Aside zanthoamides E and F and ZP-amide E, other isolated compounds had higher activity than nerve growth factor (NFG) that served as standard. It was also shown that tetrahydrobungeanool (the only isolated compound that has a non-oxygenated unsaturated long fatty acid side chain) exhibited the highest activity suggesting that the side chain may have enhanced its neuritogenic activity. Surprisingly, the compounds showed no activity in the presence of NFG. Although the mechanism behind the loss of activity in the presence of NFG is not known, possible antagonistic interactions between the molecules need to be investigated. Nonetheless, the findings demonstrate that the neurite outgrowth-promoting potentials of the compounds can be harnessed in managing conditions that lead to neurodegeneration, such as AD.

Similarly, the protective effects of *Z. capense* root methanol and ethyl acetate extracts on rotenone-elicited neuronal injury in SH-SY5Y neuroblastoma cells was investigated in vitro. Pretreatment of the neuroblastoma cells with the extracts was reported to significantly reduce ROS generation while improving intracellular glutathione level comparable to minocycline, a known inhibitor of rotenone activity. However, the extracts further reduced mitochondrial membrane potential compared to rotenone-intoxicated-untreated neuroblastoma cells, suggesting that mitochondrial membrane potential may not be the best indicator of mitochondrial uncoupling because it fluctuates with energy need of cells. In addition, the extracts inhibited rotenone-induced activation of caspase-3 and subsequent apoptosis. Comparatively, methanol extract was reported to have higher neuroprotective effect than ethyl acetate extract [151]. The presence of rutaecarpine and quercetin, previously shown to be anti-apoptotic [119,152] in the methanol extract might be responsible for its neuroprotective potential.

Furthermore, in neuronal PC12 cells injured with hydrogen peroxide, two fractions of methanol extract of *Z. bungeanum* leaves led to higher viability and reduced injury as characterized by a lower amount of leaked lactate dehydrogenase from damaged neuronal membrane. The fractions contained quercitrin, afzelin and quercetin, and the most active fraction also contained hyperoside [127], but the compounds were not tested individually for the bioactivity. The molecular mechanisms of neuroprotection by the fractions also need to be defined through additional investigation.

Other potential neuro-activity have been reported for extracts and compounds from some *Z.* species. For example, Plazas et al. [11] screened nine *Zanthoxylum* species harvested from Colombia for cholinesterase inhibitory activities and *Z.* *schreberi* (J.F.Gmel.) Reynel ex C.Nelson (Syn. *Z. monophylum* (Lam.) P.Wilsom) bark extracts were the most active. Bioassay-directed fractionation led to the isolation of alkaloids such as berberine, chelerythrine and columbamine from *Z. schreberi* bark (Table 1) that demonstrated strong inhibitory activity against both acetylcholinesterase and butyrylcholinesterase with IC_50_ values of 0.11, 1.03 and 3.75 µM and 6.40, 3.53, and 2.05 µM, respectively, for berberine, chelerythrine and columbamine. The finding suggests that these compounds should be subjected to in vivo studies to assess their potential for managing AD. 

In an in-vitro study, the protective activities of syringic acid, one of the polyphenols isolated in the fruits and barks of *Z. heitzii* [153] was assessed against ischaemia/reperfusion (OGD/R) neuronal injury. Compared to injured-untreated cells, it was reported that injured neuronal cells pretreated with syringic acid were more viable and released less lactate dehydrogenase, showing that the phenolic acid protected the hippocampal neuronal cells from injury, and oxidative stress, and elevation in intracellular free calcium concentration. In addition, the compound was shown to improve mitochondrial membrane potential, suggesting potent neuroprotective effects [153]. Syringic acid offers neuroprotection by downregulating the expression of phosphorylated (p)-JNK and p-(p38). Collectively, it protects the neurons from injury by boosting antioxidant status while inhibiting oxidation, intracellular calcium release, and apoptosis (via inhibition of both JNK and p38 pathways).

### 4.3. Antidiabetic Effects of Zanthoxylum Species

Due to the traditional use of *Z.* species in treating diabetes [154], Kimani et al. [155] assessed their effects in controlling blood sugar level and in treating diabetes. The study showed that aqueous extract of *Z. chalybeum* stem bark normalized glucose level in oral glucose tolerance test in rats, and in alloxan-induced diabetic rats fed the extract for 14 days. The extract also prevented histological damages in pancreatic β-cells, suggesting that the mechanism of action may be related to stimulation of pancreatic β-cells to secret insulin and increase insulin sensitivity. The extract may have also acted via induction of glycogenesis and glycolysis, and attenuation of oxidative damage to pancreatic β-cells by its antioxidant phytochemicals [156]. Similarly, Agwaya et al. [157] showed that aqueous extract of *Z. chalybeum* root bark at 400 mg/kg restored blood sugar level of alloxan-induced diabetic rats after 28 days of daily oral administration. The extract also returned the histo-architecture of pancreatic β-cells to near-normal. The ability of the extract to induce regeneration of the pancreatic β-cells and normalize the blood glucose level suggest that it contains phytoconstituents that can induce insulin secretion, inhibit in-vivo glucose synthesis and breakdown of stored glucose, and/or increase the sensitivity of insulin to its receptor and the consequent increase in glucose uptake. Results of these investigations support the traditional use of *Z. chalybeum* in treating diabetes in Lower Eastern Province of Kenyan [158].

Karki et al. [159] reported that aqueous-alcohol extract of *Z. armatum* bark suppressed fasting blood glucose levels of streptozotocin-diabetic rats by 43% after oral consumption of 400 mg/kg b.w. for 21 days. The extract further attenuated lipid metabolism dysfunction associated with diabetes and boosted antioxidant status in the liver and kidney. However, the mechanisms of anti-hyperglycemia and anti-hyperlipidemia were not defined. In another study, Rynjah et al. [160] demonstrated that ingestion of aqueous extract of *Z. armatum* leaves dose-dependently reduced blood glucose level in both normal and alloxan-diabetic mice, suggesting both hypoglycemic and anti-diabetic properties. The extract restored the glucose level of diabetic rat to normal level and strongly suppressed the activities of enzymes that are centrally involved with hyperglycemia, including α-amylase and α/β-glucosidases, in the rats. These results show that phytochemicals in the extracts may have acted via more than one mechanism, including by increasing insulin sensitivity, protecting pancreatic β-cells, regenerating weakened pancreatic β-cells, and inhibition of carbohydrate digestion and assimilation into the blood. For instance, Alam et al. [161] found that methanol extracts of *Z. armatum* leaves, bark, and fruits inhibited α-glucosidase, a key enzyme involved in carbohydrate digestion in the small intestinal brush border for absorption, by 94%, 97%, and 84%, respectively. The extracts also demonstrated moderate anti-hyperglycemic effects on alloxan-induced diabetes in rats with the leaf extract showing highest glucose suppressing activity. The extracts also prevented hyperlipidemia associated with diabetes, suggesting that the extracts may also be used to attenuate complications associated with diabetes.

Some mechanisms have been proposed for hypoglycemic and pancreatic cell regeneration effects of *Z. chalybeum* and *Z. armatum* [155,156,157,159,160,161]. The administration of the extracts to diabetic rats inhibited ATP-dependent K^+^ channel, leading to reduced intracellular K^+^ level and concomitant opening of L-type Ca^2+^ channel leading to increased intracellular Ca^2+^ level. This causes the depolarization of the pancreatic β-cell membrane and release of insulin. Insulin binds to its receptor on cellular surfaces to induce glucose uptake through glucose transporters (GLUTs), thus lowering blood glucose level. The extracts also inhibit the activities of enzymes involved in glucose production (gluconeogenic enzymes, α-amylase and α/β-glucosidase), thus contributing to reduced blood glucose level. The extracts, by their antioxidant activities, may also prevent reactive oxygen species (ROS)-mediated β-cell damage and apoptosis as well as induce the regeneration of pancreatic β-cells. 

### 4.4. Gastroprotective Effects of Zanthoxylum Species

About 10% of the world population suffer from gastric ulcer [162], a major cause of hospitalization and thousands of deaths globally [163]. Gastric ulcer results from the alteration in the balance between offensive factors, such as gastric acid, ROS and *Helicobacter pylori*, and protective factors, including mucus secretion, prostaglandins, nitric oxide, bicarbonate ions, enzymatic, and non-enzymatic antioxidants [164,165]. Symptoms of gastric ulcers include burning or constant pain in the stomach [166]. The etiology of gastric ulcer disease is multifactorial including overconsumption of alcohol and non-steroidal anti-inflammatory drugs, stress, smoking, and *H. pylori* infection [167]. Drugs for gastric ulcer treatment are usually targeted in the control of stomach acid secretion, K^+^/H^+^-ATPase pump, *H. pylori* and resulting inflammation. Conventional anti-cholinergic drugs used in treating gastrointestinal tract disorders, particularly peptic, gastric and duodenal ulcers, exert their effects through stomach acid suppression and self-healing of gastric mucosa lining, but they also elicit side effects such as dry mouth, arrhythmias, erectile dysfunction, constipation, gynaecomastia, urinary retention and dyshematopoiesis [168]. Consequently, there is need for additional and/or alternative drugs that are clinically effective, safe, readily available, and affordable. Many medicinal plants, including some *Z.* species, are traditionally used in treating ulcers in different parts of the world. The gastroprotective properties of extracts and compounds from some of these plants are discussed below.

In both in vitro and in-vivo models, Boye et al. [169] found that the ethanol extract of *Z. zanthxyloides* root bark attenuated indomethacin-induced gastric ulceration in rats by 71% and 85% at 250 and 500 mg/kg, respectively compared to 67% by 20 mg/kg esomeprazole, an antiulcer drug. These effects were higher than those reported for a similar dose of *Z. nitidum* stem bark aqueous extract in aspirin-induced or absolute ethanol-induced ulcer in rats [170]. Moreover, Han et al. [55] documented that aqueous extracts of *Z. nitidum* roots, stems and leaves strongly suppressed ulcer index of hydrochloric acid and ethanol-provoked ulcerated rats. Differences in activity may be due to the different solvents used in extraction, phytochemical compositions of the extracts, or chemicals used to induce ulcer in the animal models. These factors may also influence the mechanism of gastroprotection of the extracts. For instance, in isolated guinea-pig ileum, the ethanol extract of *Z. zanthxyloides* root bark inhibited cholinergic receptor activity by 66% compared to 73% by atropine, nicotinic receptor activity by 45% relative to 86% by hexamethonium, and histaminic receptor activity by 60% relative to 66% by mepyramine [169]; atropine, hexamethonium and mepyramine are cholinergic, nicotinic and histaminic receptor antagonists, respectively. This implies that compounds presented in the extract exerted the gastroprotective effect by acting as a muscarinic, histaminic and cholinergic receptor antagonists. Moreover, the *Z. nitidum* stem bark aqueous extract weakly suppressed stress-generated gastric mucosal lesion by 12% and 45% compared to 80% by ranitidine hydrochloride, a histamine H2 receptor antagonist. Specific compounds in the extracts demonstrating these effects and mechanisms are largely uncharacterized. Nonetheless, total alkaloids from the extracts were proposed to be gastroprotective by increasing nitric oxide, prostaglandin E2 and antioxidant status, and by reducing lipid peroxidation, which support earlier reports that alkaloids may be responsible for the antiulcer activities of *Z. nitidum* [170,171,172].

The plant part used also influences the extent of bioactivity. For example, Qin et al. [173] reported that extract from *Z. nitidum* stem was more effective than the root extract in suppressing the amount of gastric acid and free acids in the stomach in iodoacetamide-induced ulcer in rats. Likewise, ethanol extract of *Z. rhoifolium* stem bark exhibited strong gastroprotective effect in acute gastric lesion models (absolute ethanol, HCl/ethanol and indomethacin-induced gastric lesion in mice, hypothermic-restraint stress, and ischemia/reperfusion in rats) [174]. Observed gastroprotective effects of the extract include protection of the mice mucosal lining, attenuation of ishaemia/reperfusion injury, and modulation of stress-activated gastric lesion/erosion [174]. Moreover, other indirect effects of the extracts have been proposed. Recently, *Z. nitidum* root water extract was found to dose-dependently inhibit the activity of *H. pylori* urease, which is responsible for survival of the bacteria, with IC_50_ value of 1.29 mg/mL, which was lower than the activity of acetohydroxamic acid, a known urease inhibitor (IC_50_ = 4.56 μg/mL) [56]. Thus, in addition to the gastroprotective mechanisms, *Z. nitidum* root water extract suppresses the bacterial activity that contribute to gastric mucosal erosion. The urease-inhibiting extract contained magnoflorine, sanguinarine, chelerythrine, skimmianine, and L-sesamin as the major compounds [56], which warrant further investigation for their direct or indirect links to the urease inhibitory and gastroprotective activities.

Some mechanisms have been proposed for the gastroprotective effects of *Z. rhoifolium* extract [174]. In general, the extract demonstrated strong gastroprotection in a potentially multifactorial mechanism that includes antioxidant protection of the mucosa, activation of K_ATP_ channel and increase in nitric oxide availability. Specifically, administration of the extract to a rat model of ulcer activated ATP-dependent K^+^-channel leading to influx of K^+^ and increase in intracellular K^+^ level [174]. This results in hyperpolarization of gastric mucosa membrane causing the release of mucus and bicarbonate ions (HCO_3^−^_) to buffer the corrosive effects of gastric acid. Similarly, the extract increased the activity of iNOS likely via IP3K-Akt signaling. iNOS increases the availability of nitric oxide, which activates sGC-cGMP-PKG pathway, resulting in relaxation of smooth muscles and increased blood circulation in the gastric mucosa. Nitric oxide also activates COX-2 which increases prostaglandin-E2 (PG-E2) availability. PG-E2 binds to its receptors (G-Protein-coupled receptor subtypes) to induce mucus and HCO_3^−^_secretion while reducing gastric acid secretion. By its radical scavenging activity, the extract also inhibited ROS-mediated damages to mucosal tissues and promoted mucosal tissue regeneration. Collectively, the extract protected the gastric mucosa by increasing mucus and HCO_3^−^_ secretion while reducing gastric acid release through K_ATP_ and IP3K-Akt-iNOS-COX signaling, as well as antioxidant mechanisms.

### 4.5. Hepatoprotective Effects of Zanthoxylum Species

The liver plays key roles in the biotransformation of many xenobiotics and thus is at risk of damage by toxins. In some cases, the liver converts less toxic compounds to one or more toxic metabolite(s). For survival, the liver is protected by antioxidant system but hepatoxicity may occur at high amounts of toxicants. To investigate the potential hepatoprotective effects, hepatocytes, or animals pre-treated or post-treated with natural products are administered with large doses of hepatotoxins, such as hydrogen peroxide, carbon tetrachloride (CCl_4_), paracetamol, hypoxanthine/xanthine oxidase, and glucose oxidase.

Hur et al. [175] reported that oral administration of crude methanol extract of *Z. bungeanum* leaves (250 and 500 mg/kg) and its isolated protocatechuic acid (5, 10 and 20 mg/kg) for 7 days attenuated the elevation in lipid peroxidation and aniline hydroxylase activity in bromobenzene-treated rats. The samples also normalized the activity of epoxide hydrolase isolated from liver homogenate, but showed no significant effect on the activities of aminopyrine-N-demethylase and glutathione S-transferase, which play roles in xenobiotics metabolism. These findings suggest that the crude extract and protocatechuic acid had protective effects on liver by reducing the conversion of bromobenzene to its more hepatotoxic derivative, bromobenzene-3,4-oxide, and increasing the detoxification of bromobenzene-3,4-oxide.

Similarly, Verma and Khosa [176] pretreated rats with defatted ethanolic extract of *Z. armatum* leaves for 8 days, and, thereafter, treated the rats with CCl_4_. The extract significantly prevented hepatic necrosis in the pretreated rats compared to the intoxicated and untreated rats. This protection was evidenced by the modulation of parameters of liver status in serum and histological examinations of hepatic tissues of rats that received the extract. In addition, ethyl acetate, chloroform, and methanol extracts of *Z. armatum* root were evaluated for their ability to prevent paracetamol-mediated hepatic damage in rats and results showed that methanol extract showed the highest hepatoprotective activity, which was related to its high total phenolic content compared to other extracts [177]. Activity-guided fractionation identified lupeol as the active ingredient responsible for the hepatoprotective effects of the methanol extract [177]. The hepatoprotective effects of different parts of *Z. armatum* could be linked to their strong antioxidant activities [86]. In another study, pre-feeding of rats with aqueous extract of *Z. armatum* fruit that is rich in ellagic acid, chlorogenic acid, gallic acid, chrysin, quercetin, and epicatechin for 4 days at 100 mg/kg significantly protected the liver cells from toxicity caused by paracetamol overdose [178]. This effect was characterized by significantly lower activities of transaminases in the rat serum. Paracetamol, like many xenobiotics, is first metabolized in the liver, but its metabolites include reactive radicals that attack and damage liver cells. The ability of the *Z. armatum* fruit extract to protect against liver damage corresponded to the boosting of antioxidant status (elevation in catalase and vitamin C levels in serum and non-protein thiols in liver homogenate) and prevention of lipid peroxidation (lowering of malondialdehyde, a product of lipid peroxidation in the serum) [179]. Taken together, *Z.* species extracts can be further investigated as hepatoprotective agents, although the active compounds should be isolated and characterized. This will facilitate the elucidation of the molecular mechanisms as well as their pharmacological and nutraceutical applications.

### 4.6. Lipid-Lowering Effects of Zanthoxylum Species 

The link between hyperlipidemia and cardiovascular diseases as well as side effects associated with most currently-available hypolipidemic drugs necessitate the search for compounds with lipid-lowering properties. Wu et al. [180] compared the effects of petroleum ether, ethyl acetate and n-butanol fractions of crude ethanol extract of *Z. bungeanum* fruit on cholesterol metabolism in liver cell lines (HepG2) and found out that butanol fraction gave the best activity. The authors induced hyperlipidemic condition in the cultured cells using sterols and assessed the suppressive effect of the butanol fraction. It was reported that the butanol fraction (and rutin and hyperin isolated from it) significantly decreased extracellular total cholesterol, free cholesterol, triacylglycerol (TGs) and apoB levels. The treatments also reduced HMGC-R protein level and its mRNA expression in both normal and hyperlipidemic HepG2 cells more than simvastatin, a known HMG-CoA reductase (HMGC-R) inhibitor. Conversely, the fraction increased LDL-R protein level and the expression of genes involved in reverse cholesterol transport (*CYP27A1*, *LXRa* and *ABCG1*) in both normal and hyperlipidemic HepG2 cells. To support the results of cell culture study, the butanol fraction (200 mg/kg/d for 1 month) were shown to significantly reduce total cholesterol and TGs levels more than simvastatin at 3 mg/kg/d in diet-induce obese apoE knock-out mouse. Rutin and hyperin contents of the fraction (Table 3) were proposed to be responsible for the hypolipidemic effects [180].

In addition, ethanol extract of the plant’s pericarp and its eudesmin, β-sitosterol, and sesamin-rich n-butanol fraction [123] as well as flavone-rich ethanol extract of the fruits (Table 6) modulated lipid levels in 3T3-L1 adipocytes and high fat diet-induced obese mice by inhibiting adipocyte differentiation and down-regulating the expression of adipogenic genes and proteins such as fatty acid synthase, sterol regulatory-element binding protein-1 (*SREBP-1*), peroxisome proliferator-activated regulator-γ (*PPAR-γ*), and CCAAT/enhancer binding protein-α (*CCAAT/EBP-α*) [181]. Considering that flavones such as baicalin and naringenin possess obesity-suppressing properties [182,183], the anti-obesity effects of the fruit extract may, in part, be attributed to its flavones content and aliphatic acid amides (β-, γ- and hydroxy-β-sanshool) which inhibited the activity of human acyl-CoA:cholesterol acyltransferase (ACAT) [130]. Similarly, the aqueous extract of *Z. heitzii* stem also showed anti-hyperlipidemic effect in diet-induced hypercholesterolemic rats [89].

In monosodium glutamate (MSG)-induced hypertension, a study reported that aqueous extract of *F. tessmannii* stem bark lowered lipid formation and accumulation, and normalized the cardiovascular risk index and cardiac risk ratio that were altered by MSG [130]. Going further, the aqueous extract also attenuated L-NAME-induced hypertension, dyslipidemia and cardiovascular risk by lowering serum levels of triacylglycerol and total cholesterol, and atherogenic index and coronary risk while increasing HDL level and protected kidney, liver and heart tissues from L-NAME-induced organ damages [51].

Taken together, these results proved that *Z. bungeanum* fruit and pericarp, *F. tessmannii* stem bark and *Z. heitzii* stem contain phytochemicals that exhibit hypolipidemic effect in both normal and different models of hyperglycemia by inhibiting fatty acid, cholesterol, and lipoprotein syntheses while increasing reverse cholesterol transport and uptake for breakdown, making them important candidates for the prevention and management of hyperlipidemia, atherosclerosis, hypertension, and lipid-related conditions.

### 4.7. Antihypertensive Effects of Zanthoxylum Species

Li et al. [184] conducted a study to verify the traditional use of *Z. bungeanum* leaves in managing hypertension. Exposure of isolated rat aorta, precontracted with phenylephrine (1 µM), with crude methanol extract of *Z. bungeanum* leaves and its aqueous, n-hexane, ethyl acetate and n-butanol fractions led to relaxation of the aortic rings by 96.8%, 94.6%, 48.6%, 56.5% and 99.5%, respectively. The vasorelaxation effect of the aqueous extract in precontracted rat aortic rings was lost in the absence of vascular endothelium, which implies that the effect was mediated by endothelial signaling. A non-selective NOS inhibitor, L-NAME (10 µM) abolished the vasorelaxation, thus confirming the involvement of nitric oxide. In addition, a selective soluble guanylyl cyclase (sGC) inhibitor (1H-[1,2,4]-oxadiazolo-[4,3-α]-quinoxalin-1-one at 10 µM) significantly inhibited the vasorelaxation of precontracted rat aortic rings induced by the fraction. Thus, the extract may have acted via nitric oxide-mediated activation of sGC to increase cyclic guanosine monophosphate (cGMP) level. One of the ways cGMP mediates vasorelaxation is by activating cGMP-dependent protein kinases which mediate Ca^2+^ level and subsequent contractility of rat aortic rings. It was also reported that vasorelaxation induced by the most active fraction (butanol fraction) occurred via Ca^2+^-independent mechanism and store-operated Ca^2+^ channel (SOCC)-eNOS-sGC-cGMP signaling. Moreover, wortmannin, an inhibitor of protein kinase B (Akt), an upstream signaling molecule of eNOS, partially reduced vasorelaxation effect and totally inhibited elevation in cGMP concentration in the aortic rings by the butanol fraction, indicating additional involvement of the Akt-eNOS-sGC-cGMP signaling.

In another report, Fouda et al. [51] observed that orally administered aqueous extract of *Z. gilletii* stem barks (100 and 200 mg/kg/d) for 21 days resulted in over 75% reduction in systolic and diastolic blood pressures, pulse pressure, and heart rate in L-NAME-induced hypertension in rats. It was reported that the extract reduced heart rate more than the standard drug, captopril (20 mg/kg/d). The extract also improved antioxidant status, reduced oxidative stress/lipid peroxidation level, cardiac inflammation, and necrosis associated with L-NAME treatment. L-NAME acts by limiting nitric oxide availability, resulting in impaired endothelium-dependent vasodilation and hypertension. The plant extract strongly prevented this process, showing an antihypertensive effect that may be dependent on nitric oxide signaling. Exposure to MSG in early life induces obesity, hypertension, and cardiovascular diseases via induction of endothelial dysfunctions which manifest in later life [185]. The anti-hypertensive effects of *Z. gilletii* stem bark extract was further demonstrated in its ability to significantly suppress systolic, diastolic blood and pulse pressures in normal and MSG-induced hypertensive mice via NO signaling. The stem bark extract was reported to contain alkaloids, terpenoids, saponins, mucilage, coumarin, flavonoids, gallic tannin, phlobatannin and proanthocyanidols, which have individually shown antihypertensive effects in other studies. The ability of the *Z. bungeanum* leaves and *Z. gilletii* stem bark extracts to lower blood pressure and induce vasorelaxation in both in vitro and in vivo models support the use of these species traditionally for hypertension [133], and projects these as potential source of active compounds for hypertension management.

### 4.8. Cardioprotective Effects of Zanthoxylum Species 

The myocardium is susceptible to intense damage during oxidative stress. Sihotang et al. [186] reported that ethyl acetate extract of *Z. acanthopodium* fruit at 300 mg/kg b.w. protected the myocardium of rats 8 days prior to doxorubicin treatment. The extract caused a reduction in the serum levels of cardiac tissue status markers (troponin T and creatine kinase-MB) and normalized the tissue histo-architecture. The extract was shown to contain alkaloids, flavonoids, tannin, glycosides, and saponin, which have antioxidant properties. Considering that doxorubicin generates free radicals as a mechanism by which it induces cardiomyopathy, the cardioprotection by the extract could be linked with its antioxidant phytochemicals. Earlier, crude aqueous and alcoholic extracts of *Z. bungeanum* fruits were demonstrated to improve breathing rate in cultured cardiac cells. Hydroxy-α-sanshool, hydroxy-β-sanshool, xanthoxylin, mikanin, hyperin, isoquercitrin, rutin, myricetin, myricitrin, quercitrin, and isovanillic acid isolated from the extracts also increased breathing rates in the cells while kaempferol and luteorin reduced it. In a low Ca^2+^-level-induced low breathing rate in the cardiac cells, hydroxy-β-sanshool and xanthoxylin improved breathing rate by inducing calcium uptake and modulating sarcoplasmic reticulum Na^+^/K^+^- and Ca^2+^-ATPase activities. Hyperin and quercitrin, however, showed no significant effects on the calcium uptake, and the ATPases activities, suggesting that their mechanism of action is likely Ca^2+^-independent [125].

Diabetes is a defined risk factor to cardiovascular diseases; sustained hyperglycemia and the associated metabolic dysfunctions exposes the myocardium to oxidation, leading to cardiomyopathy [117,187]. Alloxan, a known hyperglycemic agent induces diabetes by inhibition of glucose-mediated insulin secretion as well as induction of oxidative damage to pancreatic β-cells (insulin-producing cells in islets of Langerhans). These two processes prevent the availability of insulin needed for glucose uptake in the cells; hence, inducing hyperglycemia in diabetes [188]. Agwaya et al. [189] assessed the potential of aqueous extract of *Z. chalybeum* root bark in modulating diabetes-related myocardial aberrations. The extract at 400 mg/kg/d for 28 days normalized the myocardial histo-architecture. The cardioprotective mechanism has not been defined but might be linked with antioxidant and hypoglycemic phytochemicals in the extract. In addition, the cardioprotective effects of other *Z.* species should be investigated especially those species with strong antioxidant properties.

### 4.9. Anti-inflammatory and Analgesic Activities of Zanthoxylum Species

Upon stimulation, cells of the immune system and related organs ignite a cascade of reactions, the inflammatory response, towards elimination of the harmful stimuli. In some cases, the body system is stimulated to ignite immune response against its own cells/organs, a condition that is seen in many auto-immune diseases [190]. Similarly, uncontrolled inflammation causes damage to the host, resulting in diseases collectively known as inflammatory diseases such as inflammatory bowel disease, allergy, asthma, atherosclerosis and many forms of skin irritation [191]. Several drugs classified as steroidal and non-steroidal anti-inflammatory drugs are currently approved for the treatment of some inflammatory conditions [192]. Although many of these drugs are clinically effective, they also elicit side effects such as ulcer, kidney damage, cardiac diseases, and cerebrovascular accident [190,193,194,195,196]. Several mitigation strategies have been suggested, including the combination of anti-inflammatory drugs with other agents that protect cells from damage or the use of safer alternatives.

Medicinal plants with a history of anti-inflammatory activity have been traditionally used to manage inflammation-related conditions. Screening the anti-inflammatory activities of these plants and identifying their active principles might provide new class of anti-inflammatory agents. Several species of *Zanthoxylum* genus have demonstrated anti-inflammatory properties. In an in vitro study, acetone extract of *Z. capense* leaves containing moderately high total phenolic and flavonoid contents demonstrated anti-inflammatory property by inhibiting 15-lipoxygenase activity, which converts linoleic acid to leukotrienes, a class of inflammatory mediators. The extract also suppressed nitric oxide synthesis in activated RAW 264.7 macrophage cell lines by 33–86% at 3.12–32 μg/mL and exhibited strong antioxidant activities, and low toxicity against Vero Monkey kidney cell lines with IC_50_ > 1000 μg/mL [15]. Furthermore, extract of *Z. rhetsa* stem bark was reported to inhibit inflammation induced by LPS in Raw 264.7 cells by suppression of COX-2 and iNOS as well as the mRNA expression of their upstream transcriptional activator, nuclear factor (NF)-κB [195].

In some studies, the ability of *Z. zanthoxyloides* root bark to stabilize erythrocyte membrane exposed to hypotonic solution [196], and attenuate inflammation in diabetic rats has been demonstrated [197], showing that the plant root bark has beneficial effects in resolving inflammatory processes associated with diabetes. Ethyl acetate fraction of alcoholic extracts of *Z. armatum* roots and stems also demonstrated anti-inflammatory effect in different models and blocked the feeling of pains in experimentally-induced pain in mice [197]. Lignans such as eudesmin, horsfieldin, fargesin, kobusin, sesamin, asarinin, planispine A, and pinoresinol-di-3,3-dimethylallyl from root and stem of *Z. armatum* (Table 6) are reported to be responsible of the activities [198].

Additionally, Chen et al. [124] reported that extracts of *Z. nitidum* root and stem suppressed physical and histological wounds observed and pains associated with mechanical injury. The extracts significantly reduced the number of writhing in acetic acid-generated writhing test and delayed the duration of pain observation in hot plate test. These results suggest that the extracts have promising analgesic properties. Furthermore, the extracts inhibited acute inflammation generated using carrageenan (paw edema) and chronic inflammation (granuloma) induced by cotton pellets [124].

Several secondary metabolites such as coumarin derivatives (8-formylalloxanthoxyletin, avicennone, (*Z*)-avicennone, alloxanthoxyletin, avicennol, avicennol methyl ether, *cis*-avicennol methyl ether, avicennin, xanthoxyletin, luvangetin, scopoletin, and aesculetin dimethyl ether) (Table 4), benzo[*c*]phenanthridine derivatives (norchelerythrine and arnottianamide), a furoquinoline (γ-fagarine), a triterpene (β-amyrin), and steroids (β-sitosterol and stigmasterol (Table 8)) isolated from *Z. avicennae* stem bark were subjected to anti-inflammatory activity using fMet-Leu-Phe/cytochalasin B (fMLP/CB)-induced superoxide and elastase release in cultured human neutrophiles. The release of these factors in activated neutrophils has been implicated in the initiation of inflammation. Among the isolated compounds, 8-formylalloxanthoxyletin, alloxanthoxyletin, xanthoxyletin, aesculetin dimethyl ether, and γ-fagarine significantly suppressed superoxide formation (IC_50_ ≤ 7.65 μg/mL) compared to diphenyleneiodonium standard (IC_50_ = 0.53 μg/mL). Similarly, 8-formylalloxanthoxyletin, (*E*)-avicennone, alloxanthoxyletin, avicennin, and xanthoxyletin significantly suppressed elastatase release (IC_50_ ≤ 8.17 μg/mL) compared to phenylmethylsulfonyl fluoride standard (IC_50_ = 34.2 μg/mL). Based on these findings, the anti-inflammatory properties of the extract are attributable mostly to its coumarin constituents; thus, these compounds have potential for further exploration as potent anti-inflammatory agents [119].

Wang et al. [199] isolated zanthoamides A–D, bugeanumamide A, (2*E*,7*E*,9*E*)-*N*-(2-hydroxy-2-methylpropyl)-6,11-dioxo-2,7,9-dodecatrienamide, ZP-amides A-E and tetrahydrobungeanool from *Z. bungeanum* pericarps. Considering the previously reported anti-inflammatory activities of the plant part and ZP-amides A and D isolated from *Z. bungeanum* pericarps [199], the compounds were tested against nitric production by LPS-stimulated RAW264.7 macrophages. It was reported that only zanthoamide A, tetrahydrobungeanool, ZP-amides C and D, and (2*E*,7*E*,9*E*)-*N*-(2-hydroxy-2-methylpropyl)-6,11-dioxo-2,7,9-dodecatrienamide showed inhibitory activities with IC_50_ values of 48.7, 27.1, 49.8, 112.9 and 39.4 μM, respectively. Some amides from *Z. bungeanum* pericarps also demonstrated anti-inflammatory activities through the inhibition of nitric oxide synthesis (Table 7).

Based on previously reported anti-inflammatory [119,199] and antioxidant [200] activities, Wang et al. [201] examined the effects of *Z. bungeanum* seed oil on ovalbumin-induced bronchial asthma, a complex disease whose etiology is related to inflammatory and oxidative processes. In mice with bronchial asthma, orally administered seed oil at 2 g/kg/d of suppressed pulmonary injury and infiltrations of inflammatory cells caused mainly by leukocytes. The seed oil was shown to inhibit the protein levels and mRNA expression of inflammatory cytokines and chemokines and other mediators of inflammation and their receptors as well as signaling of mitogen-activated protein kinases pathway.

Considering its traditional use in relieving bone-related pain [53], Hwang et al. [63] investigated the potential application of *Z. bungeanum* in treating experimentally-generated osteoarthritis. It was reported that crude extracts of different parts of *Z. bungeanum* inhibited monosodium iodoacetate-generated inflammation as follows: stalks (65%), roots (11.8%), twigs (84.7%), fruits (72.8%) and leaves (91.6%). The leaves were further extracted with different solvents and the ethanol extract was found to inhibit arthritic inflammation in mice by 91% at 100 mg/kg b.w. The ethanol leave extract also suppressed hind paw edema formation, feeling of pain and histologically-observed tissue damages by inflammatory process more than the standard, celecoxib at 10 mg/kg. In acetic acid-generated writhings, a model of studying the central nervous system (CNS) response to pain, pretreatment of mice with graded doses of extract suppressed the number of abdominal/stomach writhings at 50 and 100 mg/kg similar to the effect of 10 mg/kg of celecoxib. In other models of CNS mediated pain, the extract at 100 mg/kg gave 74% inhibition of hot plate-generated pain and 34% and 56.8% inhibition of phases 1 and 2 of formalin-provoked pain test, respectively [63]. The extract was further reported to downregulate genes that increase feeling of pain (K^+^-gated regulated membrane protein—KCNJ6) in isolated BV-2 mouse microglia cells, suggesting that the extract inhibited feeling of pain in both peripheral and CNS likely by inhibiting K^+^-current. In sub-chronic ear edema induced by 12-O-tetradecanoylphorbol-13-acetate (TPA), orally administered extract (25, 50 and 100 mg/kg) increased ear thickness and weight, indicating edema inhibition, and downregulated the protein level and mRNA expression of iNOS and COX-2. Furthermore, the extract suppressed mRNA expression of NF-κB and its ability to activate the expression of iNOS and COX-2 in LPS-activated RAW 264.7 macrophages. When activated, NF-κB induces the translocation of p65 from the cytosol into the nucleus for the activation of iNOS and COX-2 expression. Translocation of p65 from cytosol into the nucleus was reported to be blocked by 100 mg/kg of the extract [63]. The extract also suppressed LPS-activated ROS generation in the macrophages; increase in ROS generation is one mechanism of inducing damages to cells during inflammation. Additionally, the extract was shown to affect two genes whose role in arthritis is not defined; it suppressed hypoxanthine phosphoribosyltransferase-1 gene expression and increased ribosomal protein-L8 gene expression. It is hypothesized that the two genes may be promoting and inhibiting inflammatory events in osteoarthritis, respectively, but further study is required to clarify this effect. Two flavonoids, quercetin and afzelin that have anti-inflammatory and antioxidant activities [121,122], were reported to be isolated from the extract.

In another arthritis model using Freund’s complete adjuvant (FCA)-induced rheumatoid arthritis in rats, Lei et al. [202] topically treated arthritic rats for 21 days with *Z. bungeanum*-cake-separated moxibustion, a method that is practiced in China. It was reported that the topical treatment significantly suppressed edema formation, histological markers of inflammatory tissue damages and levels of cytokines (IL-1*β* and TNF-*α*) in serum. The anti-inflammatory activities of the *Z. bungeanum* could be attributed to the lignan components that have been shown to suppress inflammation by inhibiting nitric oxide synthesis which can signal the release of inflammatory mediators [203]. Furthermore, Rahman et al. [204] reported that methanol extract of *Z. rhetsa* stem bark moderately inhibited acetic acid-generated writhings in mice by 47.82% and 58.89% at 250 and 500 mg/kg, respectively compared to 67.30% by reference drug, aspirin at 100 mg/kg. This finding demonstrates that the extract has antinociceptive property. Similarly, at 250 and 500 mg/kg doses, ethanol extract of *Z. budrunga* root bark was reported to significantly increase the latency time of response to hot plate induced pain (5.80 and 6.81 s) relative to control (3.29 s), but the analgesic activity of the extract was lower than the effect of morphine (9.60 s) [179]. Using another analgesic assay (acetic acid-induced writhings in mice), the authors reported that the extract potently suppressed the number of writhes by 64.58% and 77.43% at 250 and 500 mg/kg, respectively, relative to 81.9% by diclofenac sodium, the reference antinociceptive drug [179]. Taken together, the studies show that *Z.* species are potential sources of analgesics, anti-arthritis, anti-asthmatic, and generally anti-inflammatory agents.

### 4.10. Anti-Thrombotic Activity of Zanthoxylum Species 

In vitro, several compounds isolated from chloroform fraction of *Z. schinifolium* bark, including schinicoumarin, acetoxyaurapten, schininallylol, aurapten, collinin, (–)-acetoxycollinin and dictamnine, showed significant antiplatelet aggregatory response with collinin exhibiting the best activity in rabbit platelet aggregation induced by collagen, arachidonic acid and platelet-activating factor [205]. Similarly, among over 30 compounds isolated from *Z. schinifolium* root bark, 8-methoxyanisocoumarins H, acetoxycollinin, schinilenol, schinindiol, anisocoumarin H, platydesmine, norchelerythrine, amottianamide, tetracosyl ferulate, β-sitosterol, and 7-[(*E*)-7′-hydroxy-3′,7′-dimethylocta-2′,5′-dienyloxy] coumarin significantly inhibited the platelet aggregation [122]. Moreover, plasminogen activator inhibitor-1 (PAI-1) is a natural negative regulator of urokinase-type plasminogen activator (uPA) and tissue-type plasminogen activator (tPA) that, when over expressed, is implicated in thrombosis, fibrin clogging of organs and arteries. Yu et al. [13] reported the inhibitory effects of *Z. nitidum* var. tomentosum against PAI-1 in vitro as a potential mechanism of its traditional use in managing blood clotting and circulation disorders in Chinese medicine. This activity guided the isolation of active compound, toddalolactone, which also inhibited clot formation and induced breakdown of ferric chloride-formed clots in vitro. Similarly, toddalolactone inhibited ferric chloride-generated clot formation and pretreatment of mice with toddalolactone (1 mg/kg/d) for 14 days significantly increased bleeding time [14]. Histological assessment of arteries of thrombotic mouse pretreated with toddalolactone showed significant inhibition of thrombus compared to control. Furthermore, toddalolactone at 1 mg/kg/day for 14 days also significantly inhibited hepatic fibrosis induced by oral administration of carbon tetrachloride (150 µL/day for a week) in mice [14]. In addition, histological assessment of liver section of the mice showed strong dose-dependent inhibition of hepatic fibrosis in mice fed toddalolactone. These findings strongly demonstrate that toddalolactone is a potential candidate for preventing and managing thrombosis and other related cardiovascular diseases. 

### 4.11. Antispasmodic Activity of Zanthoxylum Species 

Traditionally, different parts of *Z. fagara* are used in Brazil to suppress muscle spasm and modulate muscle tone and contraction. Crude ethanol extract of the stem bark showed good antispasmodic activity in rat ileum in vitro [124]; however, (–)-R-geilbalansine, hyemaline, *O*-methylbalsamide, (*R)*-tembamide, *O-*methyltembamide, zanthoxyline, nitidine, lupeol, and skimmianine isolated from the extract showed weak activity [124]. The antispasmodic activities of *Z. fagara* stem bark ethanol extract is relative to that by ethanolic and aqueous extracts of *Z. rhoifolium* in rabbit duodenum [206]. Additionally, methanol extracts of *Z. armatum* fruit, bark and leaves that inhibited butyryl cholineesterase also potently relaxed precontracted rabbit jejunum strips, intestine, trachea and thoracic aortic rings, indicating spasmolytic potentials [207]. These reports generally imply that the *Z.* species are rich sources of lignans that possess muscle-relaxation properties. The biological activities of crude extracts from different parts of *Z.* species, their solvent fractions and compounds isolated from them have been summarized in Table 10.

## 5. Toxicological Aspects of *Zanthoxylum* Species

To ascertain the safety of root bark extract of *Z. chalybeum*, a common medicinal plant used in treating many diseases in Uganda, Engeu et al. [204] fed Wistar rats with 100 and 4000 mg/kg/day for one month. The researchers reported nephrotoxicity characterized by elevation in creatinine, sodium and potassium levels in serum at 4000 mg/kg. Histological examination of the intestines showed that high doses also induced early signs of intestinal tumor formation. This observation is supported by a recent study which showed that chloroform-methanol extracts of leaves, stem bark, and root bark of *Z. chalybeum* were lethal to mice at doses above 2000 mg/kg. Similarly, high dose of the crude extract was also cytotoxic to mammalian kidney epithelium cells (Vero199) in vitro. However, the toxicity was substantially diminished upon solvent fractionation, with no sign of toxicity observed for the fractions up to 5000 mg/kg. These observations collectively imply that only low doses are recommended due to safety concerns.

Despite the common traditional uses of *Z. gilletii* to enhance sexual performance in Peru, South Africa and other regions, the plant extract negatively affected male reproductive system of rats that were fed its ethanol extract for two weeks [204]. Interestingly, a combination of *Lepidium meyenii* and *Z. gilletii* improved the fertility depression induced by *Z. gilletii* in rats [211]. In another study, Ogunbolude et al. [106] reported that 150 µg/mL of ethanol extract of *Z. zanthoxyloides* stem bark induced genotoxicity and cytotoxicity in human leukocytes. Similarly, extracts of *Z. lepreurii* and *Z. zanthoxyloides* at high concentrations were toxic to normal human prostate epithelium cells (PNT2) [58,212]. Methanol extract of *Z. zanthoxyloides* root bark was also shown to be lethal at high doses by inducing convulsion in mice; the LD_50_ was recorded to be 5000 mg/kg. Histological examination of mice that died after consuming large doses of the extract showed substantial damage to the liver and kidney [20]. Umaru et al. [213] demonstrated that aqueous extract of *Z. zanthoxyloides* stem bark dose-dependently reduced bile secretion in rats fed the extracts for 3 weeks. Interestingly, the lipid levels in rats fed the extracts were lower than the control, which implies that the stem bark polar compounds disrupted lipid digestion and absorption, which require the emulsification capacity of the bile. The above findings support the previous report that ingestion of *Z. zanthoxyloides* at higher doses than the recommended value in Ugandan traditional medicine was characterized by acute stomachache and related gastrointestinal discomfort [214].

In a study to ascertain the maximum tolerable doses of *Z. heitzii* stem bark from Cameroon, Ntchapda et al. [215] fed wistar rats with scaler doses of aqueous extract (ranging between 3000 and 18,000 mg/kg) once and recorded many signs of toxicity and organ damage especially at high doses. It was reported that the maximum tolerable dose in rodents was 6000 mg/kg b.w. Despite the promising anti-thrombin activity of toddalolactone from *Z. nitidum* var. tomentosum [13] and other extracts with anti-platelet aggregatory response, caution must be taken to avoid administration of the plant materials or its isolate to people with clotting disorders to avoid worsening the conditions. Generally, it can be inferred from the reviewed studies that only low doses of extract of plants in this genus should be further investigated for therapeutic applications. Additionally, safety doses of all the *Z.* species of nutritional and medicinal importance should be established to allow consumers make evidence-based decision on the intake doses devoid of toxicity.

## 6. Conclusions and Future Prospects

There has been heightened efforts to discover natural products as safer alternatives to drugs for managing and treating human diseases. Medicinal plants that are used traditionally for managing health challenges are currently being investigated to validate the folkloric claims. Many compounds isolated from plant origin are showing a wide variety of biological activities. Specifically, over 500 secondary metabolites have been isolated from *Z.* species and promising biological activities of many of them have been demonstrated. However, the molecular mechanisms of action of most of the bioactive compounds are not known; hence, further studies are warranted to fill this knowledge gap. In addition, the active principles of many extracts of *Zanthoxylum* species are not yet known. At high doses, some *Z.* species have exhibited toxicity, inferring that only low doses should be administered in future studies. It is also worth noting that comprehensive toxicity studies on the majority of the plant species, especially in vivo are lacking. Hence, long term safety profile in animal model is required to establish the safety doses. Similarly, bioactive compounds derived from *Z.* species whose biological activities are only demonstrated in vitro should also be tested in vivo. This is because the physiological barriers may reduce the bioaccessibility and bioavailability of the compounds, thus influencing their amounts and bioactivity in target tissues. Interestingly, the similarity in the phytochemical profiles of the plant species reviewed may explain the relatedness of the species in both their traditional medicinal uses and biological activities. Compounds isolated from *Z.* species such as hesperidin, sesamin, berberine, rutaceline, nitidine, fagaronine chelerythridine, and sanguinarine among others that demonstrated significant biological activities with known mechanism of action should be subjected to clinical trials to further assess their potential as new drug and nutraceutical candidates or as pharmacophores for the development of new generation of therapeutic agents.

## Figures and Tables

**Table 1:** Ethnobotanical applications of *Zanthoxylum* species around the world.

Plant Species	Part Used	Region or Country	Traditional Uses	Refs.
*Z. nitidum* (Roxb.) DC.	S, R	Kanayatn Dayak Community, West Kalimantan, Indonesia	Boiled with water or chewed raw to prevent alcohol intoxication and heal respiratory diseases	[53]
	S	Thailand	For treating toothache and oral pathogens	[54]
	R, S, L	China and Portugal	For treating ulcer	[55,56]
*Z. zanthoxyloides* Waterman (Syn. *Fagara zanthoxyloides* Lam.	RB, S, SB, L	Nigeria	For treating rheumatism, sickle cell anemia, toothache, malaria, urinary tract infection and venereal diseases	[34,35,36,57]
	RB	Uganda	For treating elephantiasis, toothache, sexual impotence, gonorrhea, malaria, dysmenorrhea and abdominal pain	[20,21]
	S	Cote D’ Ivoire	Prepared as a decoction to relieve tooth pains and to treat infection by oral pathogens	[37]
	L	Togo	For treating wounds	[38]
	RB		For treating toothache, swellings, and worms and induce lactation post-partum	
	S, SB		For relieving pains	
	SB	Ghana	Prepared as a decoction for treating malaria	[8]
	DP	Yamboro Community of Central African Republic	For healing diseases of circulatory and respiratory systems, malaria, diabetes, and hypertension	[39]
	F	Cameroon	For managing fever, malaria, tumors and sickle cell anemia	[58]
	SB, R, L		To suppress pains, and treat arthritis, leprosy, stomachache and venereal diseases	[59,60]
	S, R	Burkina Faso	For treating sickle cell	[61]
*Z. gilletii* (De Wild) P.G. Waterman (Syn. *Z. marcrophylla* or *F. gilletii*)	RB, SB, L	Côte d’Ivoire	Root bark, stem bark and leaves are used for treating malaria stem bark is used for treating fungal infection	[40]
	B	Kenya	For treating malaria, rheumatism, cough, urinary tract infection and many kinds of pains	[44,46]
	SB	Cameroon and Madagascar	For treating microbial infection, cancer, inflammation, hypertension and related disorders	[3,51]
*Z. clava-herculis* L. (Syn. *Z. macrophyllum* Nutt.)	SB, L	Cameroon	For treating microbial infection, diabetes, and hypertension	[39]
*Z. davyi* Waterm.	L, S, R, SB	Zulu, South Africa Vhavenda, South Africa	Leaves and stem are powdered and used to dress wounds from snakebite and decoction from these parts are used in for treating severe coughs and colds Spines for treating infected wounds, leaves for chest pains, stem bark to treat boils, pleurisy and toothache while for mouth ulcers, sore throats and as an aphrodisiac	[42]
*Z. bungeanum* Maxim. (Syn. *Z. piperitum* Benn.)	L, S	China	For treating infection and bone diseases	[62,63,64,65]
	L, F, B	Japan	For treating bacterial and fungal infections	[66]
*Z. schinifolium* Siebold & Zucc.	L, R, S	Eastern Asia	For healing stomach pains, diarrhea, jaundice, all kinds of pain and cold	[67]
*Z. buesgenii* (Engl.) P.G. Waterman	L, R, S	Sierra Leone	For treating inflammatory conditions like arthritis and rheumatism as well as venereal diseases	[68]
		South-Western Cameroon	To increase libido and improve fertility in males	[69,70]
*Z. capense* Harv	L, R R B, RB	South Africa Limpopo province Zulu people	For treating abdominal pain, wounds, infections, asthma The roots are powdered with *C. laureola* root and taken as oral preparation for respiratory tract and oral infection Prepared as a decoction for treating tuberculosis	[16,17,18,19,71] [49] [50]
*Z. usambarense* (Engl.) Kokwaro	R, F, B, S, L	Kenya	For treating malaria	[46]
*Z. tessmannii* Engl	SB	Cameroon; Madagascar	For boosting libido and treating tumor, gonorrhea and other infections, swellings, erectile dysfunction, hypertension and heart diseases	[9,51,72]
*Z. chalybeum* Engl.	SB	Kenya	For treating malaria	[46]
*Z. paracanthum* Kokwaro	SB	Kenya	For treating tumor and abdominal infection	[73]
*Z. lepreurii* Guill. & Perr.,	SB	Côte D’Ivoire; Nigeria	For treating malaria and sickle cell	[14,15,35]
*Z. limonella* (Dennst.) Alston.	EO	Mexico; Thailand	In suppressing muscle spasm and as natural herbicide	[74,75]
*Z. fagara* (L.) Sarg. (Syn. Z. *affine* Kunth and *Z. hyemale* A. St.-Hil.)	AP, B	Cuba	For treating diarrhea, cardiac disorders, fever and many types of pains such as ear and muscle pain and toothaches	[47]
*Z. elephantiasis* Macfad.	AP	Cuba	For treating diarrhea, cardiac disorders, fever and many types of pains such as ear and muscle pain and toothaches	[47]
*Z. martinicense* (Lam.) DC.	AP	Cuba	For treating diarrhea, cardiac disorders, fever and many types of pains such as ear and muscle pain and toothaches	[47]
*Z. riedelianum* Engl.	L	Brazil	For treating tumors and suppressing toothache and pains	[75]
*Z. acanthopodium* DC.	AP	China	As contraceptives and in suppressing pains and in parasite control	[48]
*Z. rhetsa* DC. (Syn. *Z. budrunga* DC.)	AP	Thailand	For treating infections	[1]
*Z. americanum* Mill.	DP	USA; Canada	For treating tumors and fungal infections of skin, and respiratory, urinary, genital and gastrointestinal tract	[76,77]
Z. *madagascariense* Baker	SB	Madagascar	For treating cancer and tuberculosis	[78]
*Z. ovalifolium* (Engl.) Finkelstein	SB	Australia	For treating cancer	[79]
	F	India	bacterial and fungal infections	[80]
*Z. rhoifolium* Lam.	SB	Brazil; France	Microbial infection of the mouth, malaria, skin and wounds	[81]
*Z. tsihanimposa* H. Perrier	SB	Madagascar	Treating inflammation, skin diseases, microbial infection and malaria	[52]
*Z. schreberi* (J.F.Gmel.) Reynel ex C.Nelson (Syn. *Z. monophylum* (Lam.) P.Wilsom)	R, B	Colombia	Memory loss and related conditions	[11]
*Z. caribaeum* Lam. (Syn. *Z. chiloperone* var. angustifolium Engl.)	SB	France; Portugal	For treating cancer and swellings	[82,83]
*Z. armatum* DC. (Syn. *Z. alatum* Roxb. and *Z. planispinum* Siebold & Zucc.)	F, B, L, R	Abbottabad; Nepal	Treating oral pathogens, cough, diabetes, tumor, microbial infections	[84,85,86]
*Z. heitzii* (Aubrev. & Pellegr.) P.G. Waterman	SB	Cameroon	Treating urogenital infections, malaria, cancer, cardiopathies, and hypertension and weight management	[87,88,89]
*Z. sprucei* Engl.	SB	Peru	Treating tumor	[90]
*Z. parachanthum* Kokwaro	SB	Kenya	For treating tumors	[2]
*Z. ailanthoides* Seibold. & Zucc.	SB, S	China	For treating tumors and HIV	[91,92,93]
*Z. lemairei* (De Wild) P.G. Waterman	SB	Nigeria	For healing malaria and running stomach	[94]
*Z. tingoassuiba* A. St.-Hil.	B	Brazil	Microbial infections, inflammation, stomach ache, muscle pains and as analgesic	[95,96]
*Z. quinduense* Tul.	SB	Colombia	For treating fungal infection of humans and plants	[97]
*Z. avicennae* (Lam.) DC.	B, L	China; Korea	For treating fungal infection of humans and plants	[98]
*Z. coreanum* Nakai	L	Korea	For treating oral pathogens and cough	[84]

SB = stem bar, L = leaves, S = seeds, RB = RB, AP = Aerial part, R = whole root, F = fruits EO = essential oil; DP = all parts/different parts.

**Table 2:** Alkaloidal compounds isolated from *Zanthoxylum* species with their biological activities.

Isolated Compound	Biological Activities	Part of Plant	Refs.
**Zanthoxylum alkaloids with anti-obesity activities**			
6-Hydroxypellitorine	Anti-obesity activity	*Z. heitzii* stem bark	[111]
Heitziquinone	Anti-obesity activity	*Z. heitzii* stem bark	[111]
Isoarnottianamide	Anti-obesity activity	*Z. heitzii* stem bark	[111]
Rhoifoline B	Anti-obesity activity	*Z. heitzii* stem bark	[111]
Sylvamide	Anti-obesity activity	*Z. heitzii* stem bark	[111]
**Zanthoxylum alkaloids with antioxidant, gastroprotective and anti-inflammatory activities**			
N,N-dimethyllindicarpin	Antioxidant activity	*Z. zanthoxyloides* root bark	[105]
1,8-di-*O*-(3-methoxy-4-hydrobenzoyl)-3,6-dihydroxycyclooctane-2,7-endoperoxide	Antioxidant activity	*Z. zanthoxyloides* root bark	[105]
Fagaronine	Antioxidant activity	*Z. zanthoxyloides* root bark	[105]
Norchelerythrine	Antioxidant activity	*Z. zanthoxyloides* root bark	[105]
Trans-fagaronine	Antioxidant activity	*Z. zanthoxyloides* root bark	[105]
8-Acetonyldihydrochelerythrine	Antioxidant activity	*F. zanthoxyloides* root bark and *Z. paracanthum* stem bark	[62,73]
Myrtopsine	antioxidant activity	*Z. zanthoxyloides* fruits	[58]
Ribalinine	Antioxidant activity	*Z. zanthoxyloides* fruits	[58]
N-isobutyl-(2*E*,4*Z*)-octa-2,4-dienamide, N-isobutyl-(2*E*,4*Z*)-deca-2,4-dienamide	Antioxidant activity	*F. zanthoxyloides* root bark	[62]
Burkinabin A	Antioxidant activity	*F. zanthoxyloides* root	[112]
Burkinabin B	Antioxidant activity	*F. zanthoxyloides* root	[112]
Burkinabin C	Antioxidant activity	*F. zanthoxyloides* root	[112]
Sanguinarine	Gastroprotective activity	*Z. gilletii* bark, root and leaves and *Z. nitidum* root	[56,113]
Fagarine I	Anti-inflammatory activity	*Z. gilletii* bark, root and leaves	[44]
**Zanthoxylum alkaloids with multiple biological activities**			
Peroxysimulenoline	Anti-inflammatory and anti-platelet aggregation activities	*Z. gilletii* bark, root and leaves and *Z. austrosinense* root	[44,113]
Skimmianine	Antioxidant and chemopreventive activities	*Z. zanthoxyloides* fruits	[58]
Xanthoplanine	Anti-inflammatory and analgesic activities	*Z. bungeanum* roots	[63,65]
**Zanthoxylum alkaloids with antispasmodic and anti-thrombotic activities**			
(-)-R-Geilbalansine	Antispasmodic activity	*Z. fagara* stem bark	[114]
Hyemaline	Antispasmodic activity	*Z. fagara* stem bark	[114]
*O*-Methylbalsamide	Antispasmodic activity	*Z. fagara* stem bark	[114]
Zanthoxyline	Antispasmodic activity	*Z. fagara* stem bark	[114]
Palmatine	Anti-thrombotic activity	Different parts of *Z. bungeanum*	[115]
Chelerythrine	Anti-thrombotic activity	Different parts of *Z. bungeanum*	[115]
8--*O*--Demethylchelerythrine	Anti-thrombotic activity	Different parts of *Z. bungeanum*	[115]
*N*--Methylcanadine	Anti-thrombotic activity	Different parts of *Z. bungeanum*	[115]
*N-*Methyltetrahydrocolumbamine	Anti-thrombotic activity	Different parts of *Z. bungeanum*	[115]
10--Demethyl--magnoflorine	Anti-thrombotic activity	Different parts of *Z. bungeanum*	[115]
3--Glucoside	Anti-thrombotic activity	Different parts of *Z. bungeanum*	[115]
Magnocurarine	Anti-thrombotic activity	Different parts of *Z. bungeanum*	[115]
Isotembetarine	Anti-thrombotic activity	Different parts of *Z. bungeanum*	[115]
8--Methoxy--isotembetatrine	Anti-thrombotic activity	Different parts of *Z. bungeanum*	[115]
Magnoflorine	Anti-thrombotic activity	Different parts of *Z. bungeanum*	[115]
Simulenoline	Anti-thrombotic activity	Different parts of *Z. bungeanum*	[115]
Benzosimuline	Anti-thrombotic activity	Different parts of *Z. bungeanum*	[115]
Zanthodioline	Anti-thrombotic activity	Different parts of *Z. bungeanum*	[115]
(–)-*N*-Acetylanonanine	Anti-thrombotic activity	Different parts of *Z. bungeanum*	[115]
(–)-*N*-Acetylnornuciferine	Anti-thrombotic activity	Different parts of *Z. bungeanum*	[115]
*N*-Acetyldehydroanonaine	Anti-thrombotic activity	Different parts of *Z. bungeanum*	[115]
Benzo [*C*] phenanthridine	Anti-thrombotic activity	Different parts of *Z. bungeanum*	[115]
Decarine	Anti-thrombotic activity	Different parts of *Z. bungeanum*	[115]
Arnottianamide	Anti-thrombotic activity	Different parts of *Z. bungeanum*	[115]
7-Fagarine	Anti-thrombotic activity	Different parts of *Z. bungeanum*	[115]
Zanthosimuline	Anti-thrombotic activity	Different parts of *Z. bungeanum*	[115]
Zanthobugeanine	Anti-thrombotic activity	Different parts of *Z. bungeanum*	[115]
Huajiaosimuline	Anti-thrombotic activity	Different parts of *Z. bungeanum*	[115]
Zanthobisquinolone	Anti-thrombotic activity	Different parts of *Z. bungeanum*	[115]
Edulitine	Anti-thrombotic activity	Different parts of *Z. bungeanum*	[115]
Arborinin	Anti-thrombotic activity	Different parts of *Z. bungeanum*	[115]
Aesculetin dimethyl ether	Anti-thrombotic activity	Different parts of *Z. bungeanum*	[115]
Oxynitidine	Anti-thrombotic activity	*Z. nitidum* stem bark	[116]
Oxyavicine	Anti-thrombotic activity	*Z. nitidum* stem bark	[116]
Oxychelerythrine	Anti-thrombotic activity	*Z. nitidum* stem bark	[116]
Dihydrochelerythrine	Anti-thrombotic activity	*Z. nitidum* stem bark	[116]
6-Acetonyldihydrochelerythrine	Anti-thrombotic activity	*Z. nitidum* stem bark	[116]
Arnottianamide	Anti-thrombotic activity	*Z. nitidum* stem bark	[116]
Liriodenine	Anti-thrombotic activity	*Z. nitidum* stem bark	[116]
*N*-Acetyldehydroanonaine	Anti-thrombotic activity	*Z. nitidum* stem bark	[116]
*N*-Acetylanonaine	Anti-thrombotic activity	*Z. nitidum* stem bark	[116]
Epizanthocadinanine A	Anti-thrombotic activity	*Z. nitidum* stem bark	[116]
**Zanthoxylum alkaloids with no reported biological activity**			
Bocconoline	Not specified	*Z. davyi* stem bark	[42]
6-Hydroxydihydrochelerythrine	Not specified	*Z. davyi* stem bark	[42]
6-Methoxy-7-demethyldihydrochelerythrine	Not specified	*Z. davyi* stem bark	[42]

**Table 3:** Flavonoids and their derivatives isolated from *Zanthoxylum* species with anti-inflammatory, antioxidant, hypolipidemic, antispasmodic and cardioprotective activities.

Isolated Compound	Biological Activities	Part of Plant	Refs.
Quercitrin	Anti-inflammatory activity	*Z*. *zanthoxyloides* leaves	[103]
Afzelin	Anti-inflammatory and antioxidant activities	*Z. bungeanum* leaves	[112,117]
Datiscin	Antioxidant activity	*Z*. *zanthoxyloides* root and leave	[103]
Neohesperidin	Antioxidant activity	*Z*. *zanthoxyloides* root and stem	[103]
Eriocitrin	Antioxidant activity	*Z. zanthoxyloides* fruits	[103]
Hyperoside	Antioxidant activity	*Z*. *zanthoxyloides* root and stem	[103]
Hesperidin	Antioxidant activity	*Z*. *zanthoxyloides* root and stem	[103]
Hesperetin	Antioxidant activity	*Z. zanthoxyloides* fruits	[58]
Hyperin	Hypolipidemic activity	*Z. bungeanum* fruit	[112]
Diosmetin	Antispasmodic activity	*Z. nitidum* leaves	[118]
Vitexin	Antispasmodic activity	*Z. nitidum* leaves	[118]
Isoquercitrin	Cardioprotective activity	*Z. bungeanum* fruits	[112]
Myricetin	Cardioprotective activity	*Z. bungeanum* fruits	[112]
Myricitrin	Cardioprotective activity	*Z. bungeanum* fruits	[112]
Rutin	Hypolipidemic, antioxidant and anti-inflammatory activities	*Z. bungeanum* fruit	[112]
Quercetin	Antioxidant and chemoprotective activities	*Z*. *zanthoxyloides* leaves	[103]
Quercetin-3-*O*-glucopyranoside	Antioxidant and chemoprotective activities	*Z*. *zanthoxyloides* leaves	[103]

**Table 4:** Coumarins and their derivatives isolated from *Zanthoxylum* species with anti-inflammatory, antioxidant, antispasmodic and anti-thrombotic activities.

Isolated Compound	Biological Activities	Part of the Plant	Refs.
8-Formylalloxanthoxyletin	Anti-inflammatory activity	*Z. avicennae* stem bark	[119]
(*Z*)-avicennone	Anti-inflammatory activity	*Z. avicennae* stem bark	[119]
Alloxanthoxyletin	Anti-inflammatory activity	*Z. avicennae* stem bark	[119]
Avicennol	Anti-inflammatory activity	*Z. avicennae* stem bark	[119]
Avicennol methyl ether	Anti-inflammatory activity	*Z. avicennae* stem bark	[119]
cis-avicennol methyl ether	Anti-inflammatory activity	*Z. avicennae* stem bark	[119]
Avicennin	Anti-inflammatory activity	*Z. avicennae* stem bark	[119]
Xanthoxyletin	Anti-inflammatory activity	*Z. avicennae* stem bark	[119]
Luvangetin	Anti-inflammatory activity	*Z. avicennae* stem bark	[119]
Scopoletin	Anti-inflammatory activity	*Z. avicennae* stem bark	[119]
Aesculetin dimethyl ether	Anti-inflammatory activity	*Z. avicennae* stem bark	[119]
7,8,9-trimethoxycoumarin	Antioxidant activity	*Z. gilletii* root	[108]
7,8-dimethoxycoumarin	Antioxidant activity	*Z. gilletii* root	[108]
Isoscopletin	Antispasmodic activity	*Z. nitidum* leaves	[120]
Zhebeiresinol	Antispasmodic and antioxidant activities	*Z. nitidum* leaves	[120]
Tetracosyl ferulate	Anti-thrombotic activity	Different parts of *Z. bungeanum*	[115]
Schinicoumarin	Anti-thrombotic activity	*Z. schinifolium* bark	[121]
Acetoxyaurapten	Anti-thrombotic activity	*Z. schinifolium* bark	[121]
Epoxycollinin	Anti-thrombotic activity	*Z. schinifolium* bark	[121]
Schininallylol	Anti-thrombotic activity	*Z. schinifolium* bark	[121]
Schinilenol	Anti-thrombotic activity	*Z. schinifolium* bark	[121]
Schinindiol	Anti-thrombotic activity	*Z. schinifolium* bark	[121]
Aurapten	Anti-thrombotic activity	*Z. schinifolium* bark	[121]
Collinin	Anti-thrombotic activity	*Z. schinifolium* bark	[121]
Epoxyaurapten	Anti-thrombotic activity	*Z. schinifolium* bark	[121]
Hydrangetin	Anti-thrombotic activity	*Z. schinifolium* bark	[121]
Umbelliferone	Anti-thrombotic activity	*Z. schinifolium* bark	[121]
Acetoxycollinin	Anti-thrombotic activity	*Z. schinifolium* bark	[121]
8-methoxyanisocoumarin H	Anti-thrombotic activity	*Z. schinifolium* root bark	[122]
Anisocoumarin H	Anti-thrombotic activity	*Z. schinifolium* root bark	[122]
Platydesmine	Anti-thrombotic activity	*Z. schinifolium* root bark	[122]
Amottianamide	Anti-thrombotic activity	*Z. schinifolium* root bark	[122]
Tetracosyl ferulate	Anti-thrombotic activity	*Z. schinifolium* root bark	[122]
7-[(*E*)-7′-hydroxy-3′,7′-dimethylocta-2′,5′-dienyloxy] coumarin	Anti-thrombotic activity	*Z. schinifolium* root bark	[122]

**Table 5:** Terpenes and their derivatives isolated from *Zanthoxylum* species with anti-obesity, antioxidant, anti-thrombotic and hepatoprotective activities.

Isolated Compound	Biological Activities	Part of Plant	Refs.
Isobauerenol	Anti-obesity activity	*Z. heitzii* stem bark	[112]
Limonene	Antioxidant activity	*Z. armatum* leaves essential oil	[123]
Germacrene D	Antioxidant activity	*Z. zanthoxyloides* root and stem bark	[104]
Myrcene	Antioxidant activity	*Z. gilletti* leaves	[109]
4′-(4”-hydroxy-3”-methylbutyloxy)-2-phenylethanol	Antioxidant activity	*F. zanthoxyloides* root bark	[62]
Cymene	Antioxidant activity	*Z. armatum* leaves essential oil	[123]
α-Copaene	Antioxidant activity	*Z. armatum* leaves essential oil	[123]
γ-Terpinene	Antioxidant activity	*Z. armatum* leaves essential oil	[123]
Bornylacetate	Antioxidant activity	*Z. armatum* leaves essential oil	[123]
Camphene	Antioxidant activity	*Z. armatum* leaves essential oil	[123]
Linalool	Antioxidant activity	*Z. armatum* leaves essential oil	[123]
β-Ocimene	Antioxidant activity	*Z. armatum* leaves essential oil	[123]
*trans*caryophyllene	Antioxidant activity	*Z. armatum* leaves essential oil	[123]
Germacrene	Antioxidant activity	*Z. armatum* leaves essential oil	[123]
α-Terpinolene	Antioxidant activity	*Z. armatum* leaves essential oil	[123]
β-Amyrenone	Anti-thrombotic activity	Different parts of *Z. bungeanum*	[115]
β-Amyrin	Anti-thrombotic activity	Different parts of *Z. bungeanum*	[115]
Friedelin	Anti-thrombotic activity	*Z. schinifolium* bark	[122]
Lupeol	Hepatoprotective effect	*Z. armatum* root	[86]

**Table 6:** Lignans and their derivatives isolated from *Zanthoxylum* species with anti-inflammatory, antispasmodic, anti-thrombotic, antioxidant, cardioprotective and antiulcer activities.

Isolated Compound	Biological Activities	Part of Plant	Refs.
Zanthpodocarpins A and B,	Anti-Inflammatory activity	*Z. bungeanum* barks	[63,65]
Eudesmin	Anti-inflammatory activity	*Z. bungeanum* barks	[63,65]
(1R,2R,5R,6S)-2-(3,4-Dimethoxyphenyl)-6-(3,4-dihydroxyphenyl)-3,7-ioxabicyclo[3.3.0]octane	Anti-inflammatory activity	*Z. bungeanum* barks	[63,65]
Magnone A	Anti-inflammatory activity	*Z. bungeanum* barks	[63,65]
*rel*-(1R,5R,6S)-6-(4-hydroxy-3-Methoxyphenyl)-3,7-dioxabicyclo[3.3.0]octan-2-one	Anti-inflammatory activity	*Z. bungeanum* barks	[63,65]
Dimethoxysamin	Anti-inflammatory activity	*Z. bungeanum* barks	[63,65]
Zanthpodocarpins C-H	Anti-inflammatory activity	*Z. bungeanum* barks	[63,65]
Fargesin	Anti-inflammatory activity	*Z. armatum* root and stem	[124]
Kobusin	Anti-inflammatory activity	*Z. armatum* root and stem	[124]
Planispine A	Anti-inflammatory activity	*Z. armatum* root and stem	[124]
Pinoresinol-di-3,3-dimethylallyl	Anti-inflammatory activity	*Z. armatum* root and stem	[124]
Pinoresinol	Antispasmodic activity	*Z. nitidum* leaves	[120]
Medioresinol	Antispasmodic activity	*Z. nitidum* leaves	[120]
Piperitol-3,3-dimethylallyl ether	Anti-thrombotic activity	*Z. nitidum* stem bark	[110]
(+)-Sesamin	Antioxidant and chemopreventive activities	*Z. zanthoxyloides* fruits	[58]
Hydroxy-α-sanshool	Cardioprotective activity	*Z. bungeanum* fruits	[125]
hydroxy-β-sanshool	Cardioprotective activity	*Z. bungeanum* fruits	[125]
L-sesamin	Antiulcer activity	*Z. nitidum* root	[56]

**Table 7:** Amides isolated from *Zanthoxylum* species and their biological activities.

Isolated Compound	Class of Compound	Biological Activities	Part of Plant	Refs.
**Zanthoxylum amides with anti-inflammatory, antioxidant, antispasmodic, hypolipidemic and neuroprotective activities**				
Zanthoamide A	Alkylamide	Anti-inflammatory activity	*Z. bungeanum* pericarps	[126]
Zanthoamide B	Alkylamide	Anti-inflammatory activity	*Z. bungeanum* pericarps	[126]
Zanthoamide C	Alkylamide	Anti-inflammatory activity	*Z. bungeanum* pericarps	[126]
Zanthoamide D	Alkylamide	Anti-inflammatory activity	*Z. bungeanum* pericarps	[126]
Bugeanumamide A	Alkylamide	Anti-inflammatory activity	*Z. bungeanum* leaves	[127,128]
Tessmamide	Aromatic amide	Antioxidant activity	*Z. gilletii* root	[108]
Robustin	Aromatic amide	Antioxidant activity	*Z. gilletii* root	[108]
Integrifoliodiol	Aromatic amide	Antioxidant activity	*Z. gilletii* root	[108]
Lupenone	Aromatic amide	Antioxidant activity	*Z. gilletii* root	[108]
Zanthoamide G	Alkalimide	Antioxidant activity	*Z. zanthoxyloides* fruits	[58]
Zanthoamide H	Alkalimide	Antioxidant activity	*Z. zanthoxyloides* fruits	[58]
Zanthoamide I	Alkalimide	Antioxidant activity	*Z. zanthoxyloides* fruits	[58]
(*R)*-tembamide	Benzamide	Antispasmodic activity	*Z. fagara* stem bark	[129]
*O-*methyltembamide	Benzamide	Antispasmodic activity	*Z. fagara* stem bark	[129]
4-methoxyquinolin-2-one	Cyclic amide	Antispasmodic activity	*Z. nitidum* leaves	[120]
β-, γ- and hydroxy-β-sanshool	Amide	Hypolipidemic effects	*Z. bungeanum* fruit	[130]
zanthoamide E and F	Alkyamide	Neuroprotective activity	*Z. bungeanum* pericarps	[126]
ZP-amide A-E	Alkyamide	Neuroprotective activity	*Z. bungeanum* pericarps	[126]
Tetrahydrobungeanool	Alkyamide	Neuroprotective activity	*Z. bungeanum* pericarps	[126]
(2*E*,7*E*,9*E*)-*N*-(2-hydroxy-2-methylpropyl)-6,11-dioxo-2,7,9-dodecatrienamide	Alkyamide	Neuroprotective activity	*Z. bungeanum* pericarps	[126]

**Table 8:** Phytosterols and their derivatives, phenol and phenolic acids, tannins, fatty acids, phenylpropanoids and steroid isolated from *Zanthoxylum* species with their biological activities.

Isolated Compound	Biological Activities	Part of Plant	Refs.
**Zanthoxylum phytosterols and their derivatives**			
Stigmasterol	Antioxidant activities	*Z. paracanthum* stem bark	[74,131]
β-sitosterol	Antioxidant and anti-thrombotic properties	*Z. budrunga* leaves and different parts of *Z. bungeanum*	[33,108,115,132]
β-sitostenone	Antioxidant and anti-thrombotic activities	Different parts of *Z. bungeanum*	[115]
**Zanthoxylum phenylpropanoids**			
4′-(3′′-methylbut-2′′-enyloxy)-3-phenylpropanol	Antioxidant activity	*F. zanthoxyloides* root bark	[62]
N-trans-coumaroyl tyramine	Antispasmodic activity	*Z. nitidum* leaves	[120]
**Zanthoxylum phenol and phenolic acids**			
2-Methoxy-4-hydroxylphenyl-1-O-α-L-rhamnopyranosyl-(1′′→6′)-β-D-glucopyranoside	Anti-inflammatory activity	*Z. armatum* stem extract	[116]
Cuspidiol	Antioxidant activity	*F. zanthoxyloides* root bark	[62]
Dihydrocusidiol	Antioxidant activity	*F. zanthoxyloides* root bark	[62]
Caffeic acid	Antioxidant activity	*Z. zanthoxyloides* stem bark	[106]
Chlorogenic acid	Antioxidant activity	*Z. zanthoxyloides* stem bark	[106]
Hydrocuspidiol	Antioxidant activity	*F. zanthoxyloides* root bark	[62]
Zanthoxylum tannins			
1H-[1,2,4]-oxadiazolo-[4,3-α]-quinoxalin-1-one	Anti-hypertensive activity	*Z. bungeanum* leaves	[133]
**Zanthoxylum long-chain fatty acids**			
Hexadecanoic acid	Antioxidant activity	*Z. zanthoxyloides* root and stem bark	[103]
**Zanthoxylum quinolones**			
Flindersine	Anti-thrombotic activity	*Z. nitidum* stem bark	[110]
4-methoxy-1-methyl-2-quinolone	Anti-thrombotic activity	*Z. nitidum* stem bark	[110]
4-methoxy-1-methyl-2(1H)-quinolinone	Not specified	*Z. davyi* stem bark	[42]

**Table 9:** Other chemicals isolated from *Zanthoxylum* species and their biological activities.

Isolated Compound	Class of Compound	Biological Activities	Part of Plant	Refs.
Atanine	Alanine derivative	Antioxidant activity	*Z. zanthoxyloides* fruits	[58]
115kDa glycoproteins	Glycoprotein	Anti-inflammatory activity	*Z. bungeanum* leaves	[134]
Isoplatydesmine	Aromatic compounds	Antioxidant activity	*Z. zanthoxyloides* fruits	[58]
*N*-methylatanine	Carboxylic acid	Antioxidant activity	*Z. zanthoxyloides* fruits	[58]
*N*-Benzoyltyramine methyl ether	Dimethyl ether	Antioxidant activity	*Z. gilletii* root	[108]
Decanal	Medium-chain aldehydes	Antioxidant activity	*Z. zanthoxyloides* root and stem bark	[105]
4-(methylamino)-benzoic acid	Phenolic acid	Antioxidant and antiulcer activities	*Z. syncarpum* branches	[135]
N-benzoyl-L-Phenylalaninol	Phenylalanine derivative	Antispasmodic activity	*Z. nitidum* leaves	[120]
4-methoxy-1-methyl-2-quinolone	Quinolone	Anti-thrombotic activity	*Z. nitidum* stem bark	[110]
Isovanillic acid	Phenolic acid	Cardioprotective activity	*Z. bungeanum* fruits	[125]
4-methoxy-1-methyl-2(1H)-quinolinone)	Quinolone	Not specified	*Z. davyi* stem bark	[42]

**Table 10:** Summary of biological activities of *Zanthoxylum* species.

Plant Species and Part Used	Test Substance	Results	Refs.
**Antioxidant properties**			
*Z. budrunga* seeds	Polyphenol-rich crude ethanol extract	Good DPPH radical scavenging effect (IC_50_ value of 82.60 μg/mL) compared to ascorbic acid (IC_50_ value of 12.58 μg/mL) and ferric reducing power relative to ascorbic acid standard	[179]
*Z. bungeanum* pericarp and seed	Magnoflorine and arbutin isolated from them	Magnoflorine and arbutin showed weak antioxidant activities	[125]
*Z. bungeanum* fruit	Hyperoside and quercitrin isolated from the fruit	Strong antioxidant and radical scavenging activities	[133]
*Z. armatum* leaves	Essential oil	Good DPPH radical scavenging activity (IC_50_ = 27 μg/mL) relative to ascorbic acid (IC_50_ = 15.0 μg/mL)	[123]
*Z. armatum* leaves	crude methanol extract, Essential oil and ethyl acetate fraction of the crude extract	Radical scavenging, ferric reducing and divalent metal chelating potentials	[85]
*Z. zanthoxyloides* fruits, leaves, stems, trunk barks, and root barks	methanol extract	High radical scavenging and ferric reducing properties	[103,180]
*Z. capense* leaves	Acetone extract	Good antioxidant and radical scavenging activities	[19]
*Z. syncarpum* Tull. Branches	crude ethanol extract, its alkaloidal fraction and 4-(methylamino)-benzoic acid isolated from it	The crude extract and its alkaloidal fraction inhibited DPPH radical scavenging activities with IC_50_ values of 140.29 μg/mL and 56.17 μg/mL, respectively relative to quercetin (IC_50_ = 4.77 μg/mL). The crude extract, the alkaloidal fraction and 4-(methylamino)-benzoic acid potently inhibited hydrochloric acid-induced corrosion; 4-(methylamino)-benzoic acid showed the best corrosion inhibition	[135]
*Z. leprieurii* stem bark	hydroethanol and aqueous	Good antioxidant activity	[15]
**Anti-diabetic effects**			
*Z. chalybeum* stem bark and root bark	Aqueous extract	Hypoglycemic activity and protected β-cells from damage	[155,157]
*Z. armatum* bark	Aqueous-ethanol extract	Anti-diabetic and hypoglycemic activities	[162]
Z. zanthoxyloides leaves	Dietary formulation	Exhibited good anti-diabetic, hepatoprotective and hypolipidemic effects against alloxan toxixity in rats	[196]
*Z. armatum* leaves	Aqueous extract	Hypoglycemic and inhibition of lipase, α-amylase and α/β-glucosidases activities	[160]
*Z. armatum* fruits, bark and leaves	methanol extract	Suppressed glucose level, increased insulin secretion via KATP channel and inhibited β-glucosidases activities (94% by bark extract, 97% by leaf extract and 84% by fruits extract	[161]
**Gatro-protective effects**			
*Z. zanthxyloides* root bark	Ethanol extract	Inhibited ulcer index by 71% and 85% at 250 and 500 mg/kg via inhibition of cholinergic, nicotinic and histaminic receptors	[135]
*Z. nitidum* stem bark	aqueous extract	inhibited ulcer by 31% and 55% at 100 and 200 mg/kg by inhibiting gastric secretion	[170]
*Z. nitidum* roots, stems and leaves	Aqueous extracts and total alkaloid extract	gastroprotective effect by increasing NO, prostaglandin E2 and antioxidant status as well as reducing lipid peroxidation	[55]
*Z. nitidum* root	aqueous extract	Inhibition of gastric acid secretion and cytotoxic against H. pylori by inhibiting its urease activity	[56]
*Z. rhoifolium* stem barks	ethanol extract	Antiulcer activity by antioxidant protection of the mucosa from free radical-mediated oxidation, activation of K_ATP_ channel and increase in NO availability	[173]
**Hepatoprotective effects**			
*Z. bungeanum* fruits	Glycoprotein	Hepatoprotective properties by mechanisms related to anti-oxidative and radical scavenging properties	[63]
*Z. bungeanum* leaves	crude methanol extract	Hepatoprotective properties by antioxidant activity and inhibition of lipid peroxidation and aniline hydroxylase activity	[174]
*Z. armatum* leaves	crude methanol extract	Inhibited hepatic damage by antioxidant mechanism	[86,175]
*Z. armatum* fruit	aqueous extract	Protected liver cells from damage by antioxidant status elevating catalase and vitamin C levels in serum and non-protein thiols in liver homogenate and lowering of malondialdehyde and liver enzyme levels in serum	[177]
**Lipid-lowering effects**			
*Z. bungeanum* fruit and pericarp	petroleum ether, Crude ethanol extract, ethyl acetate and n-butanol fractions	Hypolipidemic activity by inhibiting in vivo lipid synthesis and increasing breakdown	[118,178]
*Z. bungeanum* fruit	Ethanol extract	Inhibited lipid synthesis, transport and storage in both 3T3-L1 adipocytes and high fat diet-induced obese mouse	[179]
*Z. heitzii* stem bark	aqueous extract	Reduced lipid levels by lipid synthesis inhibition and increasing the breakdown, protected cells from hyperlipidemia damages	[89]
**Anti-hypertensive effects**			
*Z. bungeanum* leaves	crude methanol extract and its aqueous, n-hexane, ethyl acetate and n-butanol fractions	Induced vasorelaxation of smooth muscles by acting through SOCC- and Akt-eNOS-sGC-cGMP signaling in isolated aorta	[208]
Z. gilletii stem barks	aqueous extacts	Reduced systolic and diastolic blood pressures, pulse pressure and heart rate by over 75% through NO signaling	[51,148]
**Cardioprotective effects**			
*Z. acanthopodium* fruit	ethyl acetate extract	Protected the myocardium from doxorubicin-generated cardiac damage	[140]
*Z. bungeanum* fruits	crude aqueous and alcoholic extracts and hydroxy-α-sanshool, hydroxy-β-sanshool, xanthoxylin, mikanin, hyperin, isoquercitrin, rutin, myricetin, myricitrin, quercitrin and isovanillic acid isolated from them	Improved breathing rate in cultured cardiac cells by inducing calcium uptake and modulating sarcoplasmic reticulum Na^+^/K^+^- and Ca^2+^-ATPase activities	[125]
*Z. chalybeum* root bark	aqueous extract	Attenuated diabetes-related myocardial dysfunction	[157,190]
**Neuro-protective and Alzheimer′s disease modulatory effects**			
*Z. bungeanum* pericarps	crude extract and alkylamides isolated from it	Potent neuritogenic activity	[126]
*Z. capense* root	methanol and ethyl acetate extracts	Inhibition of rotenone-elicited neuronal injury in SH-SY5Y neuroblastoma cells	[151]
*Z. bungeanum* leaves	crude methanol extract and flavonoid-rich fraction	Good antioxidant and radical scavenging activities and Strongly inhibited hydrogen peroxide-induced neuronal cell damage in neuronal PC12 cells	[127]
*Z. heitzii* fruits and barks	syringic acid isolated from them	Potent neuroprotective activity against ischaemia/reperfusion (OGD/R) neuronal injury	[153]
*Z. schreberi* bark	Crude extract and berberine, chelerythrine and columbamine from it	Strongly inhibited acetylcholinesterase and butyrylcholinesterase	[11]
*Z. monophylum* bark	Crude extract and berberine, chelerythrine and columbamine isolated from it	Strongly inhibited acetylcholinesterase and butyrylcholinesterase	[11]
**Anti-inflammatory and antinociceptive effects**			
*Z. austrosinense* root	Alkaloids isolated from ethanol extract	Strongly inhibited NO production in LPS-activated mouse macrophage RAW 264.7 cells (IC_50_ values = 0.89–9.62 μM)	[114]
*Z. budrunga* seeds	crude ethanol extract	The extract at 250 and 500 mg/kg inhibited acetic acid-induced writhings in mice by 65.28% and 74.30%, respectively relative to 81.95% inhibition by diclofenac sodium that served as standard. Similarly, the extract at the same doses time and dose-dependently inhibited hot plate induced pain at latency time slightly lower than morphine that served as standard.	[203]
*Z. capense* leaves	Acetone extract	Good anti-inflammatory activity by inhibiting 15-lipoxygenase activity in vitro and suppressed nitric oxide synthesis in activated RAW 264.7 macrophage by 33–86% at 3.12–32 μg/mL	[11]
*Z. zanthoxyloides* root	aqueous extract	Strongly inhibited proteases and membrane damage induced by hypotonic solution	[195]
*Z. armatum* roots and stems	Ethanol extract and ethyl acetate fraction	Demonstrated good anti-inflammatory activities against several models of inflammation	[198]
*Z. nitidum* root and stem	Methanol extract	Suppressed feeling of both acute and chronic pain as well as inhibited both acute and chronic inflammation in both in vivo and in vitro models	[119]
*Z. rhetsa* stem bark	methanol extract	Moderately inhibited acetic acid-generated writhings in mice by 47.82% and 58.89% at 250 and 500 mg/kg, respectively compared to 67.30% by reference drug, aspirin at 100 mg/kg	[202]
*Z. budrunga* root bark	ethanol extract	At 250 and 500 mg/kg doses, the ethanol extract significantly increased the latency time of response to hot plate induced pain (5.80 and 6.81 s) relative to control (3.29 s) and morphine (9.60 s). The extract potently inhibited acetic acid-provoked writhings in mice by 64.58% and 77.43% at 250 and 500 mg/kg, respectively relative to 81.9% by diclofenac sodium	[203]
*Z. bungeanum* pericarps	ethanol extract and amides isolated from it	Good anti-inflammatory activities by inhibiting NO production by LPS-stimulated RAW264.7 macrophages	[124,197,198,199,200,201,202,203,204,205,206,207,208],
**Anti-asthmatic effect**			
*Z. bungeanum* seed	Essential oil	Inhibited nitric oxide production and suppressed pulmonary injury and infiltrations of inflammatory cells by inhibiting protein level and gene expression of inflammatory mediators and their receptors	[209]
**Anti-arthritic effects**			
different parts of *Z. bungeanum*	Ethanol extract	Inhibited monosodium idoacetate-induced osteoarthritic inflammation and pain as follows: stalks (65%), roots (11.8%), twigs (84.7%), fruits (72.8%) and leaves (91.6%) through inhibition of protein level and mRNA expression of iNOS and COX-2	[201]
*Z. bungeanum* cake	*Z. bungeanum*-cake-separated moxibustion	Inhibited Freund’s complete adjuvant-induced rheumatoid arthritis by preventing edema formation, histological markers of inflammatory damages to the tissues and levels of pro-inflammatory cytokines (IL-1β and TNF-α) in serum	[63]
**Anti-thrombotic effect**			
*Z. schinifolium* bark	Coumarins, alkaloids and triterpenoids isolated from chloroform fraction	Significantly inhibited platelet aggregatory response	[179]
*Z. schinifolium* root bark	Extract and 30 isolated compounds	Significantly inhibited collagen, arachidonic acid and platelet-activating factor-induced platelet aggregation	[122]
*Z. nitidum* var. tomentosum roots	Ethanol extract and toddalolactone isolated from it	Strongly inhibited PAI-1 and PAI-1-induced clot formation and induced breakdown of preformed clot in vitro. toddalolactone also inhibited ferric chloride-generated clot formation and hepatic necrosis and increased bleeding time in mice	[13]
**Anti-spasmodic effect**			
*Z. fagara* stem bark	Crude ethanol extract	Suppressed muscle spasm and modulate muscle tone and contraction in isolated rat ileum, in vitro	[114]
*Z. armatum* fruit, bark and leaves	Methanol extract	Inhibited butyryl cholineesterase activity by 51%, 83% and 38%, respectively and potently relaxed precontracted rabbit jejunum strips, intestine, trachea and thoracic aortic rings	[207]
*Z. rhoifolium* leaves	alcoholic and aqueous extracts	Suppressed muscle spasm and modulate muscle tone and contraction in isolated rabbit duodenum, in vitro	[208]
**Oesteoprotective effect**			
*Z. piperitum* fruit	Aqueous-ethanol extract	Exerted osteoprotective effects by inhibited the activation of c-fos/NFATc1/NF-κB pathway in osteoclast of receptor activator of nuclear factor-κB ligand (RANKL)-induced osteoporosis in ovariectomized mice	[210]

## Data Availability

Not applicable.

## Abbreviations

DPPH = 2,2′-diphenyl-1-picrylhydrazyl; IC_50_ = concentration that can inhibit 50% of the reaction; GAE = gallic acid equivalent; AD = Alzheimer’s disease; NFG = nerve growth factor; ROS = reactive oxygen species; OGD/R = oxygen glucose deprivation/re-oxygenation; JNK = c-Jun N-terminal kinases; p38 MAPK = mitogen activated protein kinase p38; GLUTs = glucose transporters; NO= nitric oxide; HCO_3^−^_ = bicarbonate; iNOS = inducible nitric oxide synthase; IP3K = inositol-3-phophate kinase; Akt = protein kinase B; SOCC = store-operated Ca^2+^ channel; eNOS = endothelial nitric oxide synthase; sGC = soluble guanylate cyclase; cGMP = cyclic guanosine monophosphate; PKG = protein kinase G; COX-2 = cyclo-oxygenase type 2; PG-E2 = prostaglandin-E2; K_ATP_ = adenosine triphosphate-dependent K^+^-channel; CCl_4_ = carbon tetrachloride; TGs = triacylglycerol; HMGC-R = 3-hydroxy-3-methyl glutaryl coenzyme A reductase; MSG= monosodium glutamate; ACAT = acyl-CoA:cholesterol acyltransferase; SREBP-1 = sterol regulatory-element binding protein-1; PPAR-γ = peroxisome proliferator-activated regulator-γ; CCAAT/EBP-α = CCAAT/enhancer binding protein-α; L-NAME = N^G^-nitro-L-arginine methyl esther; LPS = lipopolysaccharide; mRNA = messenger ribonucleic acid; NF-κB = nuclear factor-κB; fMLP/CB = fMet-Leu-Phe/cytochalasin B; CNS = central nervous system; TPA = 12-O-tetradecanoylphorbol-13-acetate; KCNJ6 = K^+^-gated regulated membrane protein; FCA = Freund’s complete adjuvant; IL = interleukins; TNF-*α* = tumor necrotic factor-*α*; uPA = urokinase-type plasminogen activator and tPA = tissue-type plasminogen activator.
